# The Theoretical Calculation of the Cu Isotope Fractionation Effect in Solution/Hydrothermal Solution Systems

**DOI:** 10.3390/molecules29112582

**Published:** 2024-05-30

**Authors:** Jixi Zhang

**Affiliations:** 1 School of Geography and Environmental Science, Guizhou Normal University, Guiyang 550001, China; jixizhang@gznu.edu.cn; 2 School of Karst Science, Guizhou Normal University/State Engineering Technology Institute for Karst Desertification, Guiyang 550001, China

**Keywords:** Cu isotope fractionation, quantum chemical calculation, solution/hydrothermal solution, solvation effect

## Abstract

Copper (Cu) is an important transition metal, and its isotopes have important applications in geology, environmental science, soil science, and other fields. Cu isotope fractionation can occur in many natural processes. However, the mechanism of Cu isotope fractionation in solution/hydrothermal solution systems is not very clear. In this study, the fractionation effects of complexes of Cu(I) and Cu(II) in solution/hydrothermal solution systems were systematically studied by means of an ab initio method based on first principles. In the simulation of an aqueous solution system, the theoretical treatment method used is the “water-droplet” method. The results show that the heavy Cu isotope (^65^Cu) enrichment capacity of the Cu-bearing complex solutions is greatly affected by the ligand types both for Cu(I) and Cu(II). For Cu(I) complex solutions, the heavy Cu isotope enrichment sequence is [Cu(HS)_2_]^−^·(H_2_O)_42_ > [Cu(HS)(H_2_O)]·(H_2_O)_42_ ≈ [Cu(HS)(H_2_S)]·(H_2_O)_42_ > [CuCl]·(H_2_O)_42_ > [CuCl_2_]^−^·(H_2_O)_42_ > [CuCl_3_]^2−^·(H_2_O)_42_. For the aqueous solutions of Cu(II) with an inorganic ligand (such as H_2_O, OH^−^, NO_3_^−^, SO_4_^2−^ and CN^−^), the order of heavy Cu isotope enrichment is as follows: [Cu(H_2_O)_6_]^2+^·(H_2_O)_42_ > [Cu(NO_3_)_2_]·(H_2_O)_42_ > [Cu(OH)_2_]·(H_2_O)_42_ > [CuSO_4_(H_2_O)_3_]·(H_2_O)_42_ > [CuNO_3_(H_2_O)_4_]^+^·(H_2_O)_42_ > [CuCN]^+^·(H_2_O)_42_. For the Cu(II) complex solutions with a halogen as ligands, the change order of 1000lnβ is [CuCl]^+^·(H_2_O)_42_ > [CuCl_2_]·(H_2_O)_42_ > [CuBr_2_]·(H_2_O)_42_ > [CuCl_3_]^−^·(H_2_O)_42_. The sequence of 1000lnβ for Cu(II) organic complex aqueous solutions is [Cu(HOC_6_H_4_COO)]^+^·(H_2_O)_42_ > [Cu(CH_3_CH_2_COO)]^+^·(H_2_O)_42_ > [Cu(COOHCOO)]^+^·(H_2_O)_42_. The calculation also found that for Cu(I) complex aqueous solutions, the difference in Cu isotope fractionation parameters (1000lnβ) between [CuCl_2_]^−^·(H_2_O)_42_ and [Cu(HS)_2_]^−^·(H_2_O)_42_ is relatively large. At 100 °C, the 1000lnβ of the two species are 1.14 and 1.55 (‰), respectively. The difference between the two could be reached up to 0.41 (‰). The Cu isotope fractionation parameter obtained with the “water droplet” method is also very different from the results of previous studies, which indicate that the Cu isotope fractionation behavior of the two is similar. At the same time, the exciting discovery is that the enrichment capacity of heavy Cu isotopes is significantly different between Cu(I) complex aqueous solutions and Cu(II) complex aqueous solutions. At 100 °C, the 1000lnβ of 6 Cu(I) complex aqueous solutions and 13 Cu(II) complex aqueous solutions ranged from 0.90 to 1.55 and 2.24 to 3.25(‰), respectively. It also shows that the REDOX reaction has a significant effect on the Cu isotope fractionation, especially in ore-forming fluids. Therefore, the ligand type is a factor that cannot be ignored when considering the mechanism of Cu isotope fractionation in solution/hydrothermal solution systems. Whether the solvation effect of an aqueous solution is considered or not has a great influence on the numerical values of the final Cu isotope fractionation factors. Hence, the solvation effect of an aqueous solution is an essential determinant in the theoretical calculation of the Cu isotope fractionation factors for Cu-bearing complex solutions.

## 1. Introduction

Copper is a very important transitional metal element, which is widely distributed in nature. Since Cu has been used as a tool to trace Cu geochemical cycling, there have been numerous studies on the Cu isotope fractionation effects [1,2,3]. There are many solid-phase Cu-bearing minerals, such as chalcopyrite, chalcocite, and bornite. The formation of various Cu-bearing minerals is closely related to the hydrothermal process. Many studies have shown that Cu isotope fractionation plays an important role in the ore-forming processes [4]. Solutions/hydrothermal solutions will play a key role in the ore-forming process. Accurately obtaining the equilibrium isotope fractionation factors of various processes can help to better understand the specific physical, chemical, and thermodynamic effects of these processes. Among them, the isotope fractionation factors of the metal–ligand (inorganic ligands, organic ligands, etc.) complexes can be obtained according to the ab initio calculation method based on first principles. Based on the basic theory of theoretical calculation geochemistry, once the simple harmonic vibration frequency of complexes is obtained, then the isotope fractionation factors can be calculated [5,6,7,8]. The acquisition of these important parameters can reveal the causes of isotope effects of various microscopic processes involved in solution/hydrothermal solution systems and provide theoretical support for the geochemical cycle of Cu isotopes.

Compared with other metal elements, Cu has obvious toxicity and fluidity, and under the right environmental conditions, it can enter the biogeochemical cycle. The decisive factor affecting the toxicity and fluidity of heavy metal Cu is the species form in the solution [9]. The phenomenon of Cu isotope fractionation is widespread in nature. The Cu isotope exchange reactions between different Cu-bearing phases (solid–liquid phase, liquid–liquid phase) will lead to the difference in Cu isotope distribution. The study of the isotope effects of Cu complexation with organic acids, such as humic acid, is also one of the hot spots for biogeochemists. Previous research results also confirmed that the Cu isotope fractionation effect does exist in the complexation process between Cu and humic acid and gave specific research data. That is, the equilibrium Cu isotope fractionation factor between solution and humic acid is ∆^65^Cu = 0.26 ± 0.11‰(2SD) [9].

In the Earth’s crust, Cu exists in valence states 0, +1, and +2 [10,11]. In this study, the Cu^+^ and Cu^2+^ are represented as Cu(I) and Cu(II), respectively. Cu(I) is relatively stable when it coordinates with HS^−^ and Cl^−^ ligands to form complexes. This is also the reason why these six Cu(I) complexes (with HS^−^ and Cl^−^ ligands) were mainly selected in this study. The application of Cu(I) and Cu(II) in aqueous solution/hydrothermal solution systems is very important. Cu has two stable isotopes, ^63^Cu and ^65^Cu, which occur in nature in an abundance of 69% and 31%, respectively [12]. Previous theoretical studies on the isotope fractionation effect of Cu(I) are relatively few, but existing studies have shown that the isotope fractionation effect of Cu(I) is widespread in hydrothermal systems [13,14,15]. When Cl^−^ is present in the solution environment, Cu(II) will exist in the form of Cu^2+^, CuCl^+^, CuCl_2_, and CuCl_3_^−^ [11]. These species can coexist polyphasically in an aqueous solution, and their concentration will change with the Cl^−^ concentration. 

An accurate understanding of the Cu isotope exchange reaction in solutions/hydrothermal solutions can provide a better understanding of the Cu geochemical cycle. However, the Cu isotope fractionation factors in different systems obtained by means of experiments can only be general data. The molecular mechanism by which these data are generated is still unclear. Since the birth of two epoch-making works at the same time, it has become a reality to obtain equilibrium isotope fractionation factors of different isotope exchange reactions from the molecular level [6,8]. Unfortunately, there are few theoretical studies on the Cu isotope fractionation effect of Cu(I) and Cu(II) aqueous/hydrothermal solution systems until now. This also greatly limits the application of Cu as a powerful tracer element in solutions/hydrothermal solutions.

Therefore, the main species of Cu(I) and Cu(II) in solutions and hydrothermal solutions were selected in this study. Previous studies have found similar Cu isotopic fractionation data for the major Cu(I) species, [CuCl_2_]^−^ and [Cu(HS)_2_]^−^, under hydrothermal conditions [13]. In hydrothermal systems, CuCl, CuCl_2_^−^, CuCl_3_^2−^, and CuCl_4_^3−^ are the dominant species forms [14]. In the selection of research systems, for the Cu(I) system, [CuCl]^0^·nH_2_O, [CuCl_2_]^−^·nH_2_O, [CuCl_3_]^2−^·nH_2_O, [Cu(HS)_2_]^−^·nH_2_O, [Cu(HS)(H_2_O)]·nH_2_O, and [Cu(HS)(H_2_S)]·nH_2_O (n = 0, 6, 12, 18, 24, 30, 36, and 42) were selected to study the isotope fractionation of Cu(I) in aqueous/hydrothermal solution systems. For the Cu(II) system, three classes of ligands were selected. That is, inorganic ligands (NO_3_^−^, H_2_O, OH^−^, CN^−^, SO_4_^2−^, etc.), organic ligands (HOC_6_H_4_COO^−^, CH_3_CH_2_COO^−^, and COOHCOO^−^), halogen-element ligands, etc., were selected to study Cu(II) isotope effects in solutions/hydrothermal solutions. The specific complex species are [Cu(HOC_6_H_4_COO)]^+^·nH_2_O, [Cu(CH_3_CH_2_COO)]^+^·nH_2_O, [Cu(COOHCOO)]^+^·nH_2_O, [CuCl]^+^·nH_2_O, [CuCl_2_]·nH_2_O, [CuCl_3_]^−^·nH_2_O, [Cu(CN)(H_2_O)_4_]^+^·nH_2_O, [Cu(H_2_O)_6_]^2+^·nH_2_O, [CuNO_3_(H_2_O)_4_]^+^·nH_2_O, [Cu(NO_3_)_2_]·nH_2_O, [Cu(OH)_2_]·nH_2_O, [CuBr_2_]·nH_2_O, and [CuSO_4_(H_2_O)_3_]·nH_2_O (n = 0, 6, 12, 18, 24, 30, 36, and 42). The isotope fractionation factors of the complexes formed by Cu ligands can be obtained with the Urey model (B-M formula) by optimizing the structure of these complexes and their aqueous solution species and calculating the harmonic vibration frequency. In theory, this is already a relatively mature theoretical calculation method that has been confirmed by a lot of theoretical research works [16,17,18].

## 2. Results

Table 1 shows the comparison of several representative Cu(I) and Cu(II) complexes in different theoretical calculation methods and experimental data. Table 2 and Table 3 show the temperature variation of the 1000lnβ of Cu-bearing aqueous solutions and inner complexes (Cu(I), Cu(II)) with different ligands under different temperature conditions.

According to the definition of Urey (1947) and Bigeleisen and Mayer (1947), the reduced partition function ratio (RPFR, that is, *β*), has a good mathematical relationship with temperature [6,8]. The following will uniformly represent the reduced partition function ratio with the parameter *β*. The mathematical expression for *β* is as follows:

(1) $$10^{3}\ln\beta =ax^{3}+bx^{2}+cx+d$$

In Equation (1), *β* is the isotope fractionation parameter (reduced partition function ratio); *a*, *b*, *c*, and *d* are coefficients corresponding to different power terms; and *x* is a function of temperature. The specific function expression of *x* is as follows:

(2) $$x=10^{6}/T^{2}$$

Through Equations (1) and (2), the *β* value can be easily obtained under any temperature conditions in the temperature range of 0–500 °C. The 1000lnβ of different Cu-bearing species (complex solutions and simple complexes) (Cu(I), Cu(II)) are given in Table 3 and Table 4. The specific values of the constant terms of the polynomial expansions are shown in Table 5. The data given in Table 5 are the optimal fitting functions in the temperature range of 0~500 °C.

**Figure 1:**
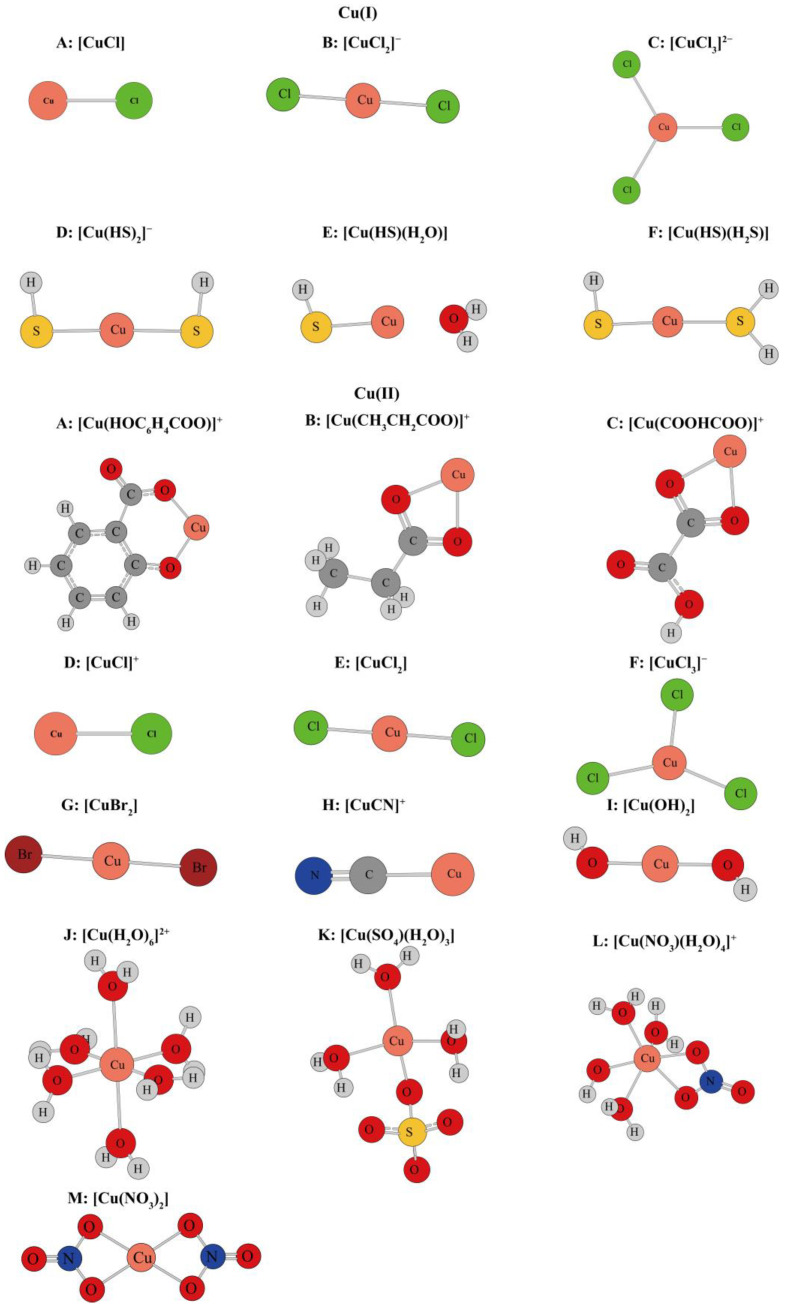
Schematic diagram of Cu(I) and Cu(II) inner layer coordination structures. The circles with different colors represent different elements, and the element symbols are marked in the corresponding positions.

**Figure 2:**
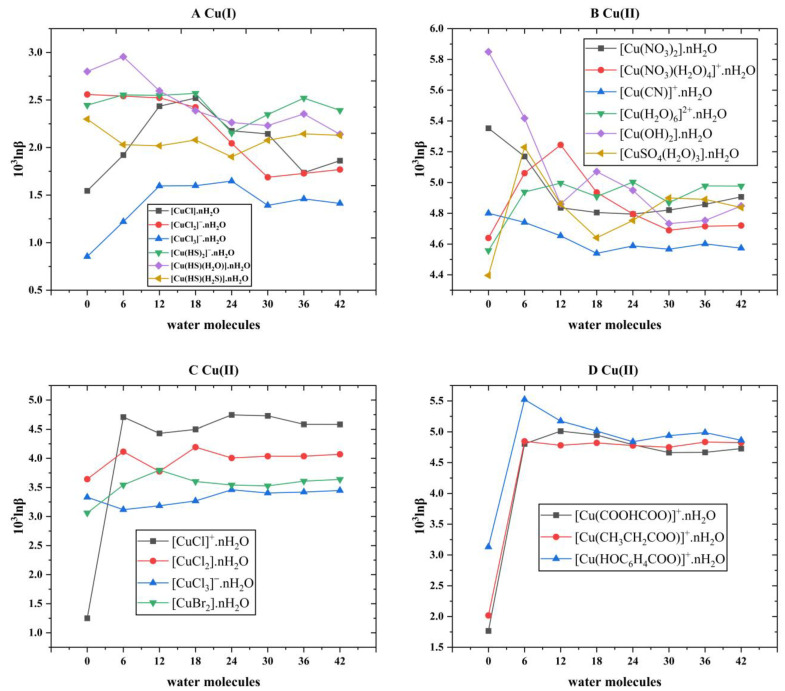
The 1000lnβs of molecular clusters of different Cu(I) and Cu(II) complex species with different water molecular numbers at 25 °C. (**A**) represents the diagram of the 1000lnβ of Cu(I) species with different water molecule numbers, and (**B**–**D**) stand for the 1000lnβ trend of Cu(II) species with different water molecule numbers.

**Figure 3:**
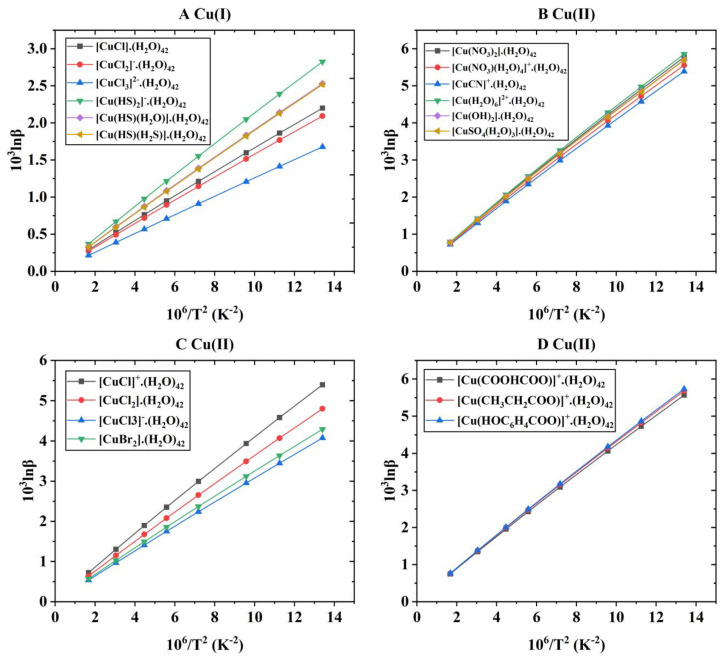
The temperature variation of the 1000lnβ of Cu(I) and Cu(II) complex solutions with different ligands. (**A**) represents different Cu(I) species, and (**B**–**D**) stand for different Cu(II) complex species. For each species, the data points from right to left represent temperatures of 0, 25, 50, 100, 150, 200, 300, and 500 °C.

**Figure 4:**
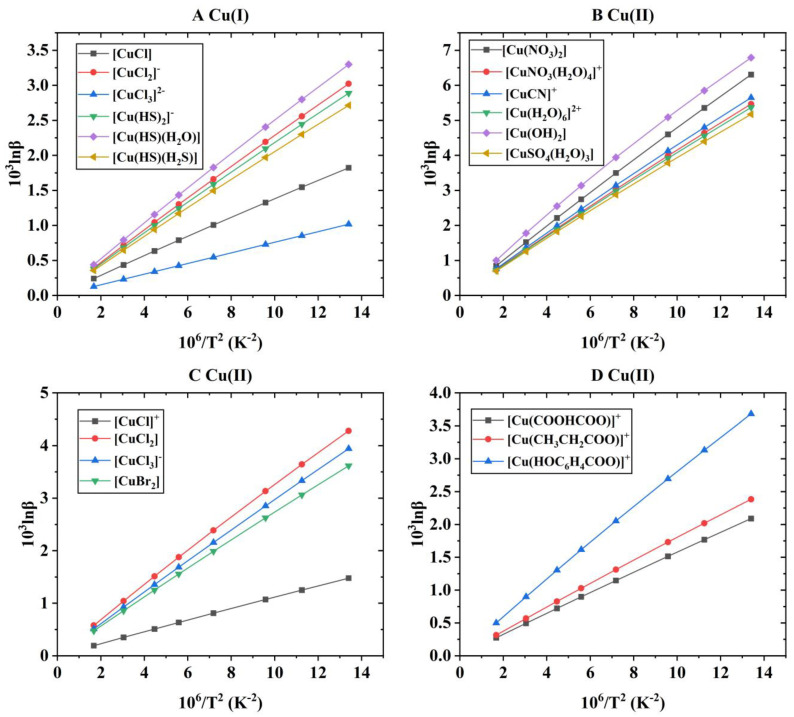
The relationship of 1000lnβ values of simple Cu(I) and Cu(II) complexes with temperature. (**A**) represents different Cu(I) species, and (**B**–**D**) stand for different Cu(II) species.

**Figure 5:**
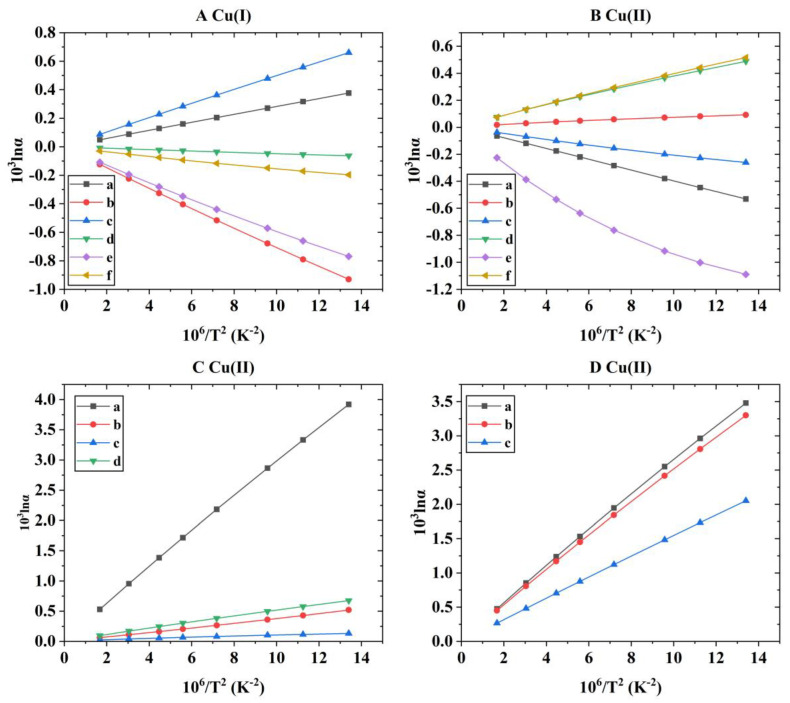
The relationship of Cu isotope fractionation factors (1000lnα) between complex solutions and simple complexes with temperature. (**A**) represents the Cu isotope fractionation factors between different Cu(I) complex solutions and simple complexes, and (**B**–**D**) stand for the Cu isotope fractionation factors between different Cu(II) complex solutions and simple complexes. In (**A**), a, b, c, d, e, and f represent the Cu isotope fractionation factors of systems [CuCl]·(H_2_O)_42_ vs. [CuCl], [CuCl_2_]^−^·(H_2_O)_42_ vs. [CuCl_2_]^−^, [CuCl_3_]^2−^·(H_2_O)_42_ vs. [CuCl_3_]^2−^, [Cu(HS)_2_]^−^·(H_2_O)_42_ vs. [Cu(HS)_2_]^−^, [Cu(HS)(H_2_O)]·(H_2_O)_42_ vs. [Cu(HS)(H_2_O)], and [Cu(HS)(H_2_S)]·(H_2_O)_42_ vs. [Cu(HS)(H_2_S)]. In (**B**), a, b, c, d, e, and f represent the Cu isotope fractionation factors of systems [Cu(NO_3_)_2_]·(H_2_O)_42_ vs. [Cu(NO_3_)_2_], [CuNO_3_(H_2_O)_4_]^+^·(H_2_O)_42_ vs. [CuNO_3_(H_2_O)_4_]^+^, [CuCN]^+^·(H_2_O)_42_ vs. [CuCN]^+^, [Cu(H_2_O)_6_]^2+^·(H_2_O)_42_ vs. [Cu(H_2_O)_6_]^2+^, [Cu(OH)_2_]·(H_2_O)_42_ vs. [Cu(OH)_2_], and [CuSO_4_(H_2_O)_3_]·(H_2_O)_42_ vs. [CuSO_4_(H_2_O)_3_], respectively. In (**C**), a, b, c, and d represent the Cu isotope fractionation factors of systems [CuCl]^+^·(H_2_O)_42_ vs. [CuCl]^+^, [CuCl_2_]·(H_2_O)_42_ vs. [CuCl_2_], [CuCl_3_]^−^·(H_2_O)_42_ vs. [CuCl_3_]^−^, and [CuBr_2_]·(H_2_O)_42_ vs. [CuBr_2_], respectively. In (**D**), a, b, and c represent the Cu isotope fractionation factors of systems [Cu(COOHCOO)]^+^·(H_2_O)_42_ vs. [Cu(COOHCOO)]^+^, [Cu(CH_3_CH_2_COO)]^+^·(H_2_O)_42_ vs. [Cu(CH_3_CH_2_COO)]^+^, and [Cu(HOC_6_H_4_COO)]^+^·(H_2_O)_42_ vs. [Cu(HOC_6_H_4_COO)]^+^, respectively.

**Figure 6:**
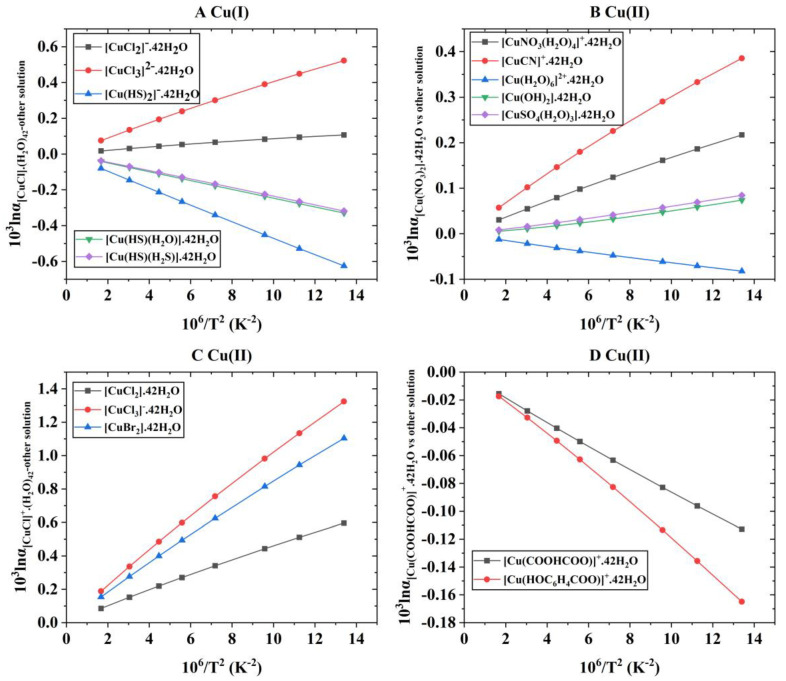
The relationship of Cu isotope fractionation factors between different Cu-bearing complex solutions with temperature. (**A**) shows the trend of Cu isotope fractionation factors between different Cu(I)-bearing complex solutions. (**B**–**D**) show the Cu isotope fractionation factors between different Cu(II)-bearing complex solutions at different temperatures. In all the graphs, the data points (from right to left) correspond to temperatures of 0, 25, 50, 100, 150, 200, 300, and 500 °C, respectively.

After the theoretical modeling of molecular clusters, the Gaussian16 software package was used to optimize the structure and calculate the harmonic vibration frequency [19]. After obtaining the harmonic vibration frequency, the reduced partition function ratio of different Cu-bearing species can be obtained with Equations (4)–(7). Figure 1 shows the coordination style of the inner structure of different Cu-bearing species. Table S1 shows the quadric parallel optimization energy of Cu complexes with different ligands in the Supplementary Materials. Figure 2 shows the variation of the 1000lnβ of molecular clusters (Cu(I) and Cu(II)) with different water molecular numbers at 25 °C. Table 4 gives constant terms of the polynomial expansions of 1000lnβ(‰) for different aqueous species. Figure 3 and Figure 4 demonstrate the temperature variation of 1000lnβ for Cu(I) and Cu(II) aqueous complexes with different ligands and simple Cu(I) and Cu(II) complexes, respectively. Figure 5 shows the relationship of the Cu isotope fractionation factors between the solution phases and the simple complexes with temperature. Table 5 and Table 6 give the Cu isotope fractionation factors (Cu(I) and Cu(II)) between solution phases and simple complexes at different temperatures and Cu isotope fractionation factors between different Cu-bearing solutions, respectively. The coefficients of the polynomial expansion of the Cu isotope fractionation factors between different complex solutions and simple complexes and between different Cu-bearing complex solutions are named Table S2 and Table S3 in the Supplementary Materials, respectively. Figure 6 shows the trend of the Cu isotope fractionation factors between different Cu-bearing solutions as a function of temperature.

**Table 1:** Comparison between different theoretical calculation methods and experimental data. Theoretical calculation methods A and B stand for the results of Seo et al. (2007) [13] and Fujii et al. (2013) [20], respectively.

Types	Bond Lengths (Å)					
	Cu(I)				Cu(II)	
	[CuCl]^0^	[CuCl_2_]^−^	[CuCl_3_]^2−^	[Cu(HS)_2_]^−^	[Cu(H_2_O)_6_]^2+^	
	Cu-Cl	Cu-Cl	Cu-Cl	Cu-S	Cu-O_1_	Cu-O_2_
This study	2.101	2.167	2.408	2.213	2.28	2.02
theoretical calculation method A	2.095	2.165	2.403	2.207		
theoretical calculation method B					2.30	2.02–2.03
experimental data	2.052 [21]	2.13 [22]			2.28 [20]	2.00 [20]

**Table 2:** The 1000lnβ of different Cu(I)-bearing and Cu(II)-bearing complex solutions at 0, 25, 50, 100, 150, 200, 300, and 500 °C.

	Temperature (°C)	0	25	50	100	150	200	300	500
Species									
Cu(I)									
[CuCl]·(H_2_O)_42_		2.19	1.86	1.59	1.21	0.94	0.76	0.52	0.28
[CuCl_2_]^−^·(H_2_O)_42_		2.09	1.76	1.51	1.14	0.89	0.71	0.49	0.27
[CuCl_3_]^2−^·(H_2_O)_42_		1.67	1.41	1.20	0.90	0.70	0.56	0.38	0.21
[Cu(HS)_2_]^−^·(H_2_O)_42_		2.82	2.39	2.04	1.55	1.21	0.97	0.66	0.37
[Cu(HS)(H_2_O)]·(H_2_O)_42_		2.52	2.13	1.83	1.38	1.08	0.87	0.59	0.33
[Cu(HS)(H_2_S)]·(H_2_O)_42_		2.51	2.12	1.82	1.37	1.07	0.86	0.59	0.32
Cu(II)									
[Cu(NO_3_)_2_]·(H_2_O)_42_		5.77	4.91	4.22	3.21	2.53	2.04	1.40	0.78
[Cu(NO_3_)(H_2_O)_4_]^+^·(H_2_O)_42_		5.56	4.72	4.06	3.09	2.43	1.96	1.35	0.75
[CuCN]^+^·(H_2_O)_42_		5.39	4.57	3.93	2.99	2.35	1.89	1.30	0.72
[Cu(H_2_O)_6_]^2+^(H_2_O)_42_		5.86	4.98	4.28	3.26	2.56	2.07	1.42	0.79
[CuCl]^+^·(H_2_O)_42_		5.40	4.58	3.94	2.99	2.35	1.89	1.30	0.72
[CuCl_2_]·(H_2_O)_42_		4.80	4.07	3.49	2.65	2.08	1.68	1.15	0.64
[CuCl_3_]^−^·(H_2_O)_42_		4.07	3.45	2.95	2.24	1.75	1.41	0.97	0.54
[CuBr_2_]·(H_2_O)_42_		4.29	3.64	3.12	2.37	1.86	1.50	1.03	0.57
[Cu(OH)_2_]·(H_2_O)_42_		5.70	4.85	4.17	3.18	2.50	2.02	1.39	0.77
[Cu(COOHCOOH)]^+^·(H_2_O)_42_		5.57	4.73	4.06	3.09	2.43	1.96	1.35	0.75
[Cu(CH_3_CH_2_COO)]^+^·(H_2_O)_42_		5.68	4.83	4.15	3.16	2.48	2.00	1.38	0.76
[Cu(HOC_6_H_4_COO)]^+^·(H_2_O)_42_		5.73	4.86	4.18	3.18	2.49	2.01	1.38	0.77
[Cu(SO_4_)(H_2_O)_3_]·(H_2_O)_42_		5.69	4.84	4.16	3.17	2.49	2.01	1.39	0.77

**Table 3:** The temperature variation of the 1000lnβ of inner complexes (Cu(I) and Cu(II)) with different ligands under different temperature conditions. The content of “[]” represents the inner coordination structure of the complex.

	Temperature (°C)	0	25	50	100	150	200	300	500
Species									
Cu(I)									
[CuCl]		1.82	1.55	1.33	1.01	0.79	0.63	0.44	0.24
[CuCl_2_]^−^		3.02	2.56	2.19	1.66	1.30	1.04	0.72	0.40
[CuCl_3_]^2−^		1.02	0.85	0.73	0.55	0.43	0.34	0.23	0.13
[Cu(HS)_2_]^−^		2.89	2.44	2.10	1.59	1.24	1.00	0.68	0.38
[Cu(HS)(H_2_O)]		3.30	2.80	2.40	1.83	1.43	1.15	0.79	0.44
[Cu(HS)(H_2_S)]		2.71	2.30	1.97	1.49	1.17	0.94	0.65	0.36
Cu(II)									
[Cu(NO_3_)_2_]		6.31	5.35	4.60	3.50	2.75	2.21	1.52	0.84
[Cu(NO_3_)(H_2_O)_4_]^+^		5.46	4.64	3.99	3.03	2.38	1.92	1.32	0.73
[CuCN]^+^		5.65	4.80	4.13	3.14	2.47	1.99	1.37	0.76
[Cu(H_2_O)_6_]^2+^		5.37	4.56	3.92	2.98	2.34	1.88	1.29	0.72
[CuCl]^+^		1.48	1.25	1.07	0.81	0.63	0.51	0.35	0.19
[CuCl_2_]		4.28	3.64	3.13	2.39	1.88	1.51	1.04	0.58
[CuCl_3_]^−^		3.94	3.33	2.85	2.16	1.69	1.35	0.93	0.51
[CuBr_2_]		3.62	3.06	2.62	1.99	1.56	1.25	0.86	0.47
[Cu(OH)_2_]		6.79	5.85	5.09	3.94	3.14	2.55	1.78	1.00
[Cu(COOHCOOH)]^+^		2.09	1.77	1.51	1.15	0.90	0.72	0.49	0.27
[Cu(CH_3_CH_2_COO)]^+^		2.38	2.02	1.73	1.31	1.03	0.83	0.57	0.31
[Cu(HOC_6_H_4_COO)]^+^		3.68	3.13	2.69	2.05	1.62	1.30	0.90	0.50
[Cu(SO_4_)(H_2_O)_3_]		5.17	4.40	3.78	2.88	2.26	1.82	1.25	0.70

**Table 4:** The polynomial expansion coefficients of 1000lnβ (‰) for different Cu(I) and Cu(II) complex species according to Equation (1). (H_2_O)_42_ represents 42 water molecules that were added to the outer layers of the complexes, i.e., the solvation effect is taken into account.

	Coefficients	a	b	c	d
Species					
Cu(I)					
[CuCl]·(H_2_O)_42_		8.8731 × 10^−6^	−8.9619 × 10^−4^	1.7445 × 10^−1^	6.3391 × 10^−4^
[CuCl_2_]^−^·(H_2_O)_42_		4.8850 × 10^−6^	−6.1887 × 10^−4^	1.6351 × 10^−1^	3.4897 × 10^−4^
[CuCl_3_]^2−^·(H_2_O)_42_		1.7512 × 10^−6^	−2.7831 × 10^−4^	1.2851 × 10^−1^	2.1363 × 10^−4^
[Cu(HS)_2_]^−^·(H_2_O)_42_		6.9430 × 10^−6^	−9.8807 × 10^−4^	2.2272 × 10^−1^	2.3775 × 10^−4^
[Cu(HS)(H_2_O)]·(H_2_O)_42_		7.8718 × 10^−6^	−8.9365 × 10^−4^	1.9919 × 10^−1^	5.3897 × 10^−4^
[Cu(HS)(H_2_S)]·(H_2_O)_42_		5.4942 × 10^−6^	−7.5608 × 10^−4^	1.9695 × 10^−1^	3.2375 × 10^−4^
[CuCl]		4.5682 × 10^−6^	−7.5276 × 10^−4^	1.4526 × 10^−1^	4.7295 × 10^−5^
[CuCl_2_]^−^		6.0135 × 10^−6^	−1.0369 × 10^−3^	2.3831 × 10^−1^	5.9667 × 10^−5^
[CuCl_3_]^2−^		6.0219 × 10^−8^	−5.3906 × 10^−5^	7.6549 × 10^−2^	1.0912 × 10^−7^
[Cu(HS)_2_]^−^		6.3388 × 10^−6^	−1.0069 × 10^−3^	2.2784 × 10^−1^	1.0440 × 10^−4^
[Cu(HS)(H_2_O)]		1.2316 × 10^−5^	−1.6046 × 10^−3^	2.6532 × 10^−1^	2.1774 × 10^−4^
[Cu(HS)(H_2_S)]		6.7653 × 10^−6^	−1.0290 × 10^−3^	2.1507 × 10^−1^	1.0880 × 10^−4^
Cu(II)					
[Cu(NO_3_)_2_]		3.4536 × 10^−5^	−3.3387 × 10^−3^	5.0881 × 10^−1^	2.2326 × 10^−3^
[Cu(NO_3_)(H_2_O)_4_]^+^		2.9116 × 10^−5^	−2.8825 × 10^−3^	4.4105 × 10^−1^	1.3720 × 10^−3^
[CuCN]^+^		3.3608 × 10^−5^	−3.2563 × 10^−3^	4.5892 × 10^−1^	2.1177 × 10^−3^
[Cu(H_2_O)_6_]^2+^		2.5416 × 10^−5^	−2.7630 × 10^−3^	4.3295 × 10^−1^	7.8647 × 10^−4^
[CuCl]^+^		2.4464 × 10^−6^	−4.8443 × 10^−4^	1.1625 × 10^−1^	2.0312 × 10^−5^
[CuCl_2_]		2.0937 × 10^−5^	−2.5141 × 10^−3^	3.4937 × 10^−1^	3.2586 × 10^−4^
[CuCl_3_]^−^		5.5308 × 10^−6^	−1.1281 × 10^−3^	3.0821 × 10^−1^	4.5520 × 10^−5^
[CuBr_2_]		7.0621 × 10^−6^	−1.2608 × 10^−3^	2.8550 × 10^−1^	6.6567 × 10^−5^
[Cu(OH)_2_]		1.4317 × 10^−4^	−9.7179 × 10^−3^	6.1070 × 10^−1^	5.4551 × 10^−3^
[Cu(COOHCOOH)]^+^		8.2807 × 10^−6^	−7.6525 × 10^−4^	1.6457 × 10^−1^	6.6464 × 10^−4^
[Cu(CH_3_CH_2_COO)]^+^		1.0411 × 10^−5^	−1.0043 × 10^−3^	1.8932 × 10^−1^	5.6713 × 10^−4^
[Cu(HOC_6_H_4_COO)]^+^		3.1919 × 10^−5^	−2.4572 × 10^−3^	3.0175 × 10^−1^	1.7156 × 10^−3^
[Cu(SO_4_)(H_2_O)_3_]		3.3183 × 10^−5^	−3.0106 × 10^−3^	4.2027 × 10^−1^	2.1138 × 10^−3^
[Cu(NO_3_)_2_]·(H_2_O)_42_		4.1814 × 10^−5^	−3.4885 × 10^−3^	4.6982 × 10^−1^	3.1179 × 10^−3^
[Cu(NO_3_)(H_2_O)_4_]^+^ (H_2_O)_42_		4.0737 × 10^−5^	−3.2940 × 10^−3^	4.5120 × 10^−1^	3.2766 × 10^−3^
[CuCN]^+^ (H_2_O)_42_		3.4484 × 10^−5^	−2.9083 × 10^−3^	4.3460 × 10^−1^	3.2864 × 10^−3^
[Cu(H_2_O)_6_]^2+^(H_2_O)_42_		4.4401 × 10^−5^	−3.6211 × 10^−3^	4.7723 × 10^−1^	3.4596 × 10^−3^
[CuCl]^+^ (H_2_O)_42_		3.5134 × 10^−5^	−2.9826 × 10^−3^	4.3617 × 10^−1^	2.7235 × 10^−3^
[CuCl_2_] (H_2_O)_42_		2.6456 × 10^−5^	−2.3114 × 10^−3^	3.8432 × 10^−1^	2.2532 × 10^−3^
[CuCl_3_]^−^ (H_2_O)_42_		1.5882 × 10^−5^	−1.5604 × 10^−3^	3.2191 × 10^−1^	1.4476 × 10^−3^
[CuBr_2_] (H_2_O)_42_		2.3327 × 10^−5^	−1.9949 × 10^−3^	3.4273 × 10^−1^	2.0328 × 10^−3^
[Cu(OH)_2_] (H_2_O)_42_		4.5740 × 10^−5^	−3.7295 × 10^−3^	4.6680 × 10^−1^	3.4371 × 10^−3^
[Cu(COOHCOOH)]^+^ (H_2_O)_42_		3.8027 × 10^−5^	−3.1905 × 10^−3^	4.5119 × 10^−1^	2.8230 × 10^−3^
[Cu(CH_3_CH_2_COO)]^+^ (H_2_O)_42_		3.9108 × 10^−5^	−3.2751 × 10^−3^	4.6054 × 10^−1^	3.0062 × 10^−3^
[Cu(HOC_6_H_4_COO)]^+^ (H_2_O)_42_		3.4751 × 10^−5^	−2.9986 × 10^−3^	4.6155 × 10^−1^	2.4027 × 10^−3^
[Cu(SO_4_)(H_2_O)_3_] (H_2_O)_42_		4.5256 × 10^−5^	−3.6418 × 10^−3^	4.6494 × 10^−1^	3.3710 × 10^−3^

**Table 5:** Cu isotope fractionation factors (Cu(I) and Cu(II)) between complex solutions and simple complexes (in the form of 1000lnα (‰)).

	Temperature (°C)	0	25	50	100	150	200	300	500
Species									
Cu(I)									
[CuCl]·(H_2_O)_42_ vs. [CuCl]		0.38	0.32	0.27	0.20	0.16	0.13	0.09	0.05
[CuCl_2_]^−^·(H_2_O)_42_ vs. [CuCl_2_]^−^		−0.93	−0.79	−0.68	−0.52	−0.40	−0.33	−0.22	−0.12
[CuCl_3_]^2−^·(H_2_O)_42_ vs. [CuCl_3_]^2−^		0.66	0.56	0.48	0.36	0.28	0.23	0.16	0.09
[Cu(HS)_2_]^−^·(H_2_O)_42_ vs. [Cu(HS)_2_]^−^		−0.06	−0.05	−0.05	−0.04	−0.03	−0.02	−0.02	−0.01
[Cu(HS)(H_2_O)]·(H_2_O)_42_ vs. [Cu(HS)(H_2_O)]		−0.77	−0.66	−0.57	−0.44	−0.35	−0.28	−0.19	−0.11
[Cu(HS)(H_2_S)]·(H_2_O)_42_ vs. [Cu(HS)(H_2_S)]		−0.20	−0.17	−0.15	−0.12	−0.09	−0.08	−0.05	−0.03
Cu(II)									
[Cu(NO_3_)_2_]·(H_2_O)_42_ vs. [Cu(NO_3_)_2_]		−0.53	−0.45	−0.38	−0.28	−0.22	−0.18	−0.12	−0.06
[Cu(NO_3_)(H_2_O)_4_]^+^·(H_2_O)_42_ vs. [Cu(NO_3_)(H_2_O)_4_]^+^		0.09	0.08	0.07	0.06	0.05	0.04	0.03	0.02
[CuCN]^+^·(H_2_O)_42_ vs. [CuCN]^+^		−0.26	−0.23	−0.20	−0.16	−0.12	−0.10	−0.07	−0.04
[Cu(H_2_O)_6_]^2+^·(H_2_O)_42_ vs. [Cu(H_2_O)_6_]^2+^		0.49	0.42	0.36	0.28	0.23	0.19	0.13	0.07
[CuCl]^+^·(H_2_O)_42_ vs. [CuCl]^+^		3.92	3.33	2.87	2.18	1.72	1.38	0.95	0.53
[CuCl_2_]·(H_2_O)_42_ vs. [CuCl_2_]		0.52	0.43	0.36	0.27	0.20	0.16	0.11	0.06
[CuCl_3_]^−^·(H_2_O)_42_ vs. [CuCl_3_]^−^		0.13	0.12	0.10	0.08	0.07	0.05	0.04	0.02
[CuBr_2_]·(H_2_O)_42_ vs. [CuBr_2_]		0.68	0.58	0.50	0.38	0.30	0.24	0.17	0.10
[Cu(OH)_2_]·(H_2_O)_42_ vs. [Cu(OH)_2_]		−1.09	−1.00	−0.92	−0.76	−0.64	−0.53	−0.39	−0.23
[Cu(COOHCOOH)]^+^·(H_2_O)_42_ vs. [Cu(COOHCOOH)]^+^		3.48	2.96	2.55	1.95	1.53	1.24	0.85	0.47
[Cu(CH_3_CH_2_COO)]^+^·(H_2_O)_42_ vs. [Cu(CH_3_CH_2_COO)]^+^		3.30	2.81	2.42	1.84	1.45	1.17	0.81	0.45
[Cu(HOC_6_H_4_COO)]^+^·(H_2_O)_42_ vs. [Cu(HOC_6_H_4_COO)]^+^		2.05	1.73	1.48	1.12	0.88	0.70	0.48	0.27
[Cu(SO_4_)(H_2_O)_3_]·(H_2_O)_42_ vs. [Cu(SO_4_)(H_2_O)_3_]		0.52	0.44	0.38	0.29	0.23	0.19	0.13	0.07

**Table 6:** Cu isotope fractionation factors between different Cu-bearing complex solutions (in the form of 1000lnα (‰)).

	Temperature (°C)	0	25	50	100	150	200	300	500
Species									
Cu(I)									
[CuCl]·(H_2_O)_42_ vs. [CuCl_2_]^−^·(H_2_O)_42_		0.11	0.09	0.08	0.07	0.05	0.04	0.03	0.02
[CuCl]·(H_2_O)_42_ vs. [CuCl_3_]^2−^·(H_2_O)_42_		0.52	0.45	0.39	0.30	0.24	0.19	0.13	0.08
[CuCl]·(H_2_O)_42_ vs. [Cu(HS)_2_]^−^·(H_2_O)_42_		−0.63	−0.53	−0.45	−0.34	−0.27	−0.21	−0.15	−0.08
[CuCl]·(H_2_O)_42_ vs. [Cu(HS)(H_2_O)]·(H_2_O)_42_		−0.33	−0.28	−0.24	−0.18	−0.14	−0.11	−0.08	−0.04
[CuCl]·(H_2_O)_42_ vs. [Cu(HS)(H_2_S)]·(H_2_O)_42_		−0.32	−0.27	−0.23	−0.17	−0.13	−0.10	−0.07	−0.04
Cu(II)									
[Cu(NO_3_)_2_]·(H_2_O)_42_ vs. [CuNO_3_(H_2_O)_4_]^+^·(H_2_O)_42_		0.22	0.19	0.16	0.12	0.10	0.08	0.05	0.03
[Cu(NO_3_)_2_]·(H_2_O)_42_ vs. [CuCN]^+^·(H_2_O)_42_		0.39	0.33	0.29	0.23	0.18	0.15	0.10	0.06
[Cu(NO_3_)_2_]·(H_2_O)_42_ vs. [Cu(H_2_O)_6_]^2+^·(H_2_O)_42_		−0.08	−0.07	−0.06	−0.05	−0.04	−0.03	−0.02	−0.01
[Cu(NO_3_)_2_]·(H_2_O)_42_ vs. [Cu(OH)_2_]·(H_2_O)_42_		0.07	0.06	0.05	0.03	0.02	0.02	0.01	0.01
[Cu(NO_3_)_2_]·(H_2_O)_42_ vs. [CuSO_4_(H_2_O)_3_]·(H_2_O)_42_		0.08	0.07	0.06	0.04	0.03	0.02	0.02	0.01
[CuCl]^+^·(H_2_O)_42_ vs. [CuCl_2_]·(H_2_O)_42_		0.60	0.51	0.44	0.34	0.27	0.22	0.15	0.09
[CuCl]^+^·(H_2_O)_42_ vs. [CuCl_3_]^−^·(H_2_O)_42_		1.32	1.13	0.98	0.76	0.60	0.49	0.34	0.19
[CuCl]^+^·(H_2_O)_42_ vs. [CuBr_2_]·(H_2_O)_42_		1.10	0.94	0.82	0.63	0.49	0.40	0.28	0.15
[Cu(COOHCOO)]^+^·(H_2_O)_42_ vs. [Cu(CH_3_CH_2_COO)]^+^·(H_2_O)_42_		−0.11	−0.10	−0.08	−0.06	−0.05	−0.04	−0.03	−0.02
[Cu(COOHCOO)]^+^·(H_2_O)_42_ vs. [Cu(HOC_6_H_4_COO)]^+^·(H_2_O)_42_		−0.16	−0.14	−0.11	−0.08	−0.06	−0.05	−0.03	−0.02

## 3. Discussion

(1) The information of the optimized structures of Cu-bearing species

It has been accepted that isotope fractionation can be affected by changes in the elemental compositions or structures of molecules and molecular clusters. Many previous studies have also confirmed such a phenomenon [6,8,23]. Therefore, it is of great theoretical and practical significance to study the isotope effects of different Cu-bearing species in solution/hydrothermal solution systems based on first principles. Table S1 shows the optimized energy data of potential Cu-bearing (Cu(I) and Cu(II)) complexes calculated in this study (see Figure 1 for the inner layer coordination). The theoretical basis set for all structural optimization and energy calculation is b3lyp/6-311+g(d). The energies of clusters with 6, 12, 18, 24, 32, 36, and 42 water molecules are shown in Table S1. To ensure the accuracy of the final calculation, four parallel calculations (represented by A, B, C, and D, respectively) were carried out when executing the energy structure optimization and the simple harmonic vibration frequency calculation. Hence, a total of more than 532 molecular clusters were optimized. The purpose of this operation is that when using the water drop method to treat solvation effects, many different conformations are used to simulate aqueous complexes, which can reduce the error that may be caused by conformational differences in a solution. The optimized structure energies of all molecular clusters are listed in Table S1 in the Supplementary Materials.

All Cu-bearing complex aqueous solution models with 42 water molecules are surrounded by multiple layers of water molecules. These water molecules are connected by hydrogen bonds to form four-, five-, and six-member rings. The four-, five-, and six-member rings in the optimized Cu-bearing complex aqueous solutions are very stable. This is also consistent with the previous research result that liquid water has a cyclic structure [24]. All the calculated harmonic frequencies have no virtual frequency, which indicates that the optimized structure is at least at a local minimum on the potential energy surface.

In the theoretical study of isotope fractionation, the change in bond length is also an important factor that directly affects the isotope fractionation effect. The theoretically optimized bond lengths of four representative Cu(I) complexes ([CuCl]^0^, [CuCl_2_]^−^, [CuCl_3_]^2−^, and [Cu(HS)_2_]^−^) and a typical Cu(II) complex ([Cu(H_2_O)_6_]^2+^) were compared with previous theoretical and experimental results to verify the rationality of the proposed method. Table 1 shows the comparison of species bond lengths between the theoretical and experimental results of this study and those of previous studies. For Cu(I) complexes such as [CuCl]^0^, [CuCl_2_]^−^, [CuCl_3_]^2−^, and [Cu(HS)_2_]^−^, the corresponding bond lengths of Cu-Cl, Cu-Cl, Cu-Cl, and Cu-S are 2.101 Å, 2.167 Å, 2.408 Å, and 2.213 Å, respectively. Corresponding previous research data were 2.095 Å [13] (2.052 Å [21]), 2.165 Å [13]) (2.13 Å [22]), 2.403 Å [13], and 2.207 Å [13], respectively. For [Cu(H_2_O)_6_]^2+^, the molecular cluster is a typical octahedral structure. Cu(II) is located in the center of the molecular cluster, and four oxygen atoms form a plane with Cu(II), and the other two oxygen atoms are located directly above and below this plane. In this sense, there are two different types of Cu-O bonds in [Cu(H_2_O)_6_]^2+^. In this study, these two different types of Cu-O bonds are named Cu-O_1_ and Cu-O_2_, respectively. For these two different types of Cu-O bonds, the results of this study and previous studies are Cu-O_1_:2.28 Å (this study), 2.30 Å [20], and 2.28 Å [25] and Cu-O_2_: 2.02 Å (this study), 2.02–2.03 Å [20] and 2.00 Å [25]. Based on the existing experimental and theoretical calculation results, it can be seen that the results of this study have a good agreement with the previous studies. This also reflects from the side that this research method is correct and reasonable.

(2) Calculated data of Cu isotope fractionation

In simulating solvation effects, the method used is the “water-droplet” method, which adds different amounts of water molecules to the periphery of the Cu-bearing complexes. In general, the more water molecules you have, the better you can simulate a real aqueous environment. However, considering the accuracy, calculation cost, and time cost in the actual calculation process, six water molecules were chosen to add as a batch around the Cu-bearing complexes until the complex (H_2_O)_42_ was obtained. As can be seen from Figure 2, whether for Cu(I) or Cu(II) complexes, when the number of water molecules exceeds 30, the change in β value is basically in a small range. In other words, when the number of water molecules exceeds 30, its β converges to a constant value. Therefore, when performing theoretical calculations, it is reasonable for us to choose a structure surrounded by 42 water molecules as the final structure to calculate the β value. 

Using Equation (1), the Cu isotope fractionation parameters (i.e., 1000lnβ) of different Cu-bearing species at 0, 25, 50, 100, 150, 200, 300, and 500 °C can be calculated. The specific β values of Cu-bearing complex solutions under the corresponding temperature conditions are shown in Table 2. For six Cu(I) aqueous solutions ([CuCl]·(H_2_O)_42_, [CuCl_2_]^−^·(H_2_O)_42_, [CuCl_3_]^2−^·(H_2_O)_42_, [Cu(HS)_2_]^−^·(H_2_O)_42_, [Cu(HS)(H_2_O)]·(H_2_O)_42_, and [Cu(HS)(H_2_S)]·(H_2_O)_42_), in the temperature range of 0–500 °C, the variation range of 1000lnβ was 2.19~0.28, 2.09~0.27, 1.67~0.21, 2.82~0.37, 2.52~0.33, and 2.51~0.32 (‰). At the same time, isotope fractionation parameters (β) considering only the inner coordination forms (without considering solvation effects) are also listed in Table 3. Figure 3A shows the relationship between the isotope fractionation parameter (1000lnβ) and temperature. As can be seen from Figure 3A, the calculated results of this study show that the change order for the 1000lnβ values of the aqueous solutions of six Cu(I) complexes is [Cu(HS)_2_]^−^·(H_2_O)_42_ > [Cu(HS)(H_2_O)]·(H_2_O)_42_ ≈ [Cu(HS)(H_2_S)]·(H_2_O)_42_ > [CuCl]·(H_2_O)_42_ > [CuCl_2_]^−^·(H_2_O)_42_ > [CuCl_3_]^2−^·(H_2_O)_42_. The largest Cu isotope fractionation (in the form of ^65/63^Cu) occurs between species [Cu(HS)_2_]^−^·(H_2_O)_42_ and [CuCl_3_]^2−^·(H_2_O)_42_. At 25 °C, the isotope fractionation factor between the two substances can reach 0.98(‰). From this change order, it can be seen that for the ligands HS^−^ and Cl^−^, the complexes with ligands of HS^−^ have a stronger ability to enrich heavy Cu isotopes than Cl^−^. For the Cu(I) complex solutions with Cl^−^ as ligands, the ability to enrich heavy isotopes gradually decreases with the increase in the amount of Cl^−^.

For Cu(II) aqueous solutions, 13 different substances were selected to study the Cu isotope fractionation effect, namely [Cu(NO_3_)_2_]·(H_2_O)_42_, [Cu(NO_3_)(H_2_O)_4_]^+^·(H_2_O)_42_, [CuCN]^+^·(H_2_O)_42_, [Cu(H_2_O)_6_]^2+^·(H_2_O)_42_, [CuCl]^+^·(H_2_O)_42_, [CuCl_2_]·(H_2_O)_42_, [CuCl_3_]^−^·(H_2_O)_42_, [CuBr_2_]·(H_2_O)_42_, [Cu(OH)_2_]·(H_2_O)_42_, [Cu(COOHCOO)]^+^·(H_2_O)_42_, [Cu(CH_3_CH_2_COO)]^+^·(H_2_O)_42_, [Cu(HOC_6_H_4_COO)]^+^·(H_2_O)_42_, and [Cu(SO_4_)(H_2_O)_3_]·(H_2_O)_42_. These species were used to simulate aqueous solution environments under different conditions. In the temperature range of 0–500 °C, the corresponding Cu isotope fractionation parameters (1000lnβ) ranged from 5.77~0.78, 5.56~0.75, 5.39~0.72, 5.86~0.79, 5.40~0.72, 4.80~0.64, 4.07~0.54, 4.29~0.57, 5.70~0.77, 5.57~0.75, 5.68~0.76, 5.73~0.77, and 5.69~0.77(‰), respectively (see Table 2 for details). At the same time, the Cu isotope fractionation parameters (1000lnβ) without considering the solvation effect of water (that is, only considering the coordination form of the inner layers) have also been systematically studied. In the same temperature range, the isotope fractionation parameters (1000lnβ) of [Cu(NO_3_)_2_], [Cu(NO_3_)(H_2_O)_4_]^+^, [CuCN]^+^, [Cu(H_2_O)_6_]^2+^, [CuCl]^+^, [CuCl_2_], [CuCl_3_]^−^, [CuBr_2_], [Cu(OH)_2_], [Cu(COOHCOO)]^+^, [Cu(CH_3_CH_2_COO)]^+^, [Cu(HOC_6_H_4_COO)]^+^, and [Cu(SO_4_)(H_2_O)_3_] were 6.31–0.84, 5.46–0.73, 5.65–0.76, 5.37–0.72, 1.48–0.19, and 4.28–0.58, 3.94~0.51, 3.62~0.47, 6.79~1.00, 2.09~0.27, 2.38~0.31, 3.68~0.50, and 5.17~0.70(‰), respectively (see Table 3 for details). Compared with the molecular clusters surrounded by water molecules on the outer side, it can be seen that the solvation effect of aqueous solutions is very significant. Taking 1000lnβ at 25 °C as an example, the values of [Cu(COOHCOO)]^+^ and [Cu(COOHCOO]^+^·(H_2_O)_42_ are 2.09 and 5.57, respectively. The difference between them could be reached up to 3.48(‰). It is well known that the isotope fractionation parameter (1000lnβ) has a good functional relationship with temperature, that is, with the increase in temperature, 1000lnβ gradually decreases. All the systems involved in this study follow this rule. Figure 3B–D show the temperature variation of the 1000lnβ of different Cu(II)-bearing complex solutions. For the Cu(II) inorganic ligand (H_2_O, OH^−^, NO_3_^−^, SO_4_^2−^ and CN^−^) complex solutions, the change sequence of 1000lnβ is as follows: [Cu(H_2_O)_6_]^2+^·(H_2_O)_42_ > [Cu(NO_3_)_2_]·(H_2_O)_42_ > [Cu(OH)_2_]·(H_2_O)_42_ > [CuSO_4_(H_2_O)_3_]·(H_2_O)_42_ > [CuNO_3_(H_2_O)_4_]^+^·(H_2_O)_42_ > [CuCN]^+^·(H_2_O)_42_ (Figure 3B). At 25 °C, the 1000lnβ of these six Cu(II) complex solutions varied from 4.57 to 4.98, with a maximum difference of 0.41(‰). For Cu(II) complex solutions with halogen elements as ligands, the variation in 1000lnβ is [CuCl]^+^·(H_2_O)_42_ > [CuCl_2_]·(H_2_O)_42_ > [CuBr_2_]·(H_2_O)_42_ > [CuCl_3_]^−^·(H_2_O)_42_ (Figure 3C). At 25 °C, the variation range of the 1000lnβ of these four Cu(II) complex solutions was 3.45~4.58, and the maximum difference was 1.13(‰). In nature, Cu(II) is one of the important components of many organisms. To investigate the Cu isotope fractionation effect in organic life activities, three organic ligands (HOC_6_H_4_COO^−^, CH_3_CH_2_COO^−^, and COOHCOO^−^) were selected to study this process. The results showed that the change sequence of 1000lnβ for these three Cu(II) organic complex aqueous solutions was [Cu(HOC_6_H_4_COO)]^+^·(H_2_O)_42_ > [Cu(CH_3_CH_2_COO)]^+^·(H_2_O)_42_ > [Cu(COOHCOO)]^+^·(H_2_O)_42_ (Figure 3D). At 25 °C, the 1000lnβ of the three Cu(II) organic complex solutions varied from 5.57 to 5.73, and the maximum difference was 0.16(‰). Compared with Cu(II) inorganic ligand aqueous solutions, the isotope fractionation effect between Cu(II) organic complex aqueous solutions is much smaller. To facilitate the acquisition of the 1000lnβ of Cu(I) and Cu(II) complex (solutions) at any temperature, the polynomial expansion coefficient terms of 1000lnβ for the research systems involved in this study are given in detail in Table 4. 

If the solvation effect is not considered (only the inner coordination structure is considered), the Cu isotope fractionation parameters of these six Cu(I) complexes are as follows: [Cu(HS)(H_2_O)] > [CuCl_2_]^−^ > [Cu(HS)_2_]^−^ > [Cu(HS)(H_2_S)] > [CuCl] > [CuCl_3_]^2−^. This change order is inconsistent with that when solvation effects are considered (Figure 4A). For the inorganic ligand complex of Cu(II), the change order of 1000lnβ is [Cu(OH)_2_] > [Cu(NO_3_)_2_] > [CuCN]^+^ > [CuNO_3_(H_2_O)_4_]^+^ > [Cu(H_2_O)_6_]^2+^ > [CuSO_4_(H_2_O)_3_] (see Figure 4B). At 25 °C, the variation range of 1000lnβ was 5.17–6.79 (‰). The sequence of the 1000lnβ of the Cu(II) complexes with halogen as ligands is [CuCl_2_] > [CuCl_3_]^−^ > [CuBr_2_] > [CuCl]^+^ (see Figure 4C). At 25 °C, the range of 1000lnβ for these four species is 1.25~3.64. The sequence of the 1000lnβ of the three Cu(II) organic complexes is [Cu(HOC_6_H_4_COO)]^+^ > [Cu(CH_3_CH_2_COO)]^+^ > [Cu(COOHCOO)]^+^ (see Figure 4D). Although the size order does not change compared to aqueous solution species, the numerical difference between each species is huge. At 25 °C, the variation range of 1000lnβ for these three species is 2.09~3.68 (‰).

(3) solvation effect on the Cu isotope fractionation

In order to better understand the solvation effect on the isotope fractionation of Cu(I) and Cu(II) complexes, the Cu isotope fractionation factors between different Cu-bearing aqueous solutions and simple complexes were systematically studied. Water is the most important driving force in the migration of elements. For the Cu(I) species, [CuCl]·(H_2_O)_42_ is a more enriched heavy Cu isotope than [CuCl], and the equilibrium Cu isotope fractionation factor varies from 0.38 to 0.05 at the temperature range of 0~500 °C. At 100 °C, the Cu isotope fractionation factor (1000lnα (‰); similarly hereinafter) between [CuCl]·(H_2_O)_42_ and [CuCl] is 0.20 (‰). For the system of [CuCl_2_]^−^·(H_2_O)_42_ vs. [CuCl_2_]^−^, at the temperature range of 0~500 °C, the range of the Cu isotope fractionation factor between these two species is −0.93~−0.12. At 100 °C, the isotope fractionation factor between [CuCl_2_]^−^·(H_2_O)_42_ and [CuCl_2_]^−^ is −0.52, and the isotope fractionation effect is very significant. However, for Cu-bearing species with HS^−^ ligands, complex solutions will deplete heavy isotopes compared with simple complexes. To be specific, the ranges of Cu isotope fractionation factors for the systems of [Cu(HS)_2_]^−^·(H_2_O)_42_ vs. [Cu(HS)_2_]^−^, [Cu(HS)(H_2_O)]·(H_2_O)_42_ vs. [Cu(HS)(H_2_O)]., and [Cu(HS)(H_2_S)]·(H_2_O)_42_ vs. [Cu(HS)(H_2_S)] are −0.06~−0.01, −0.77~−0.11, and −0.20~−0.03 (0~500 °C). For the system [Cu(HS)_2_]^−^·(H_2_O)_42_ vs. [Cu(HS)_2_]^−^, the difference in the heavy isotope enrichment capacity between the two systems is limited, that is, the solvation effect is not very obvious. For the other two systems, ([Cu(HS)(H_2_O)]·(H_2_O)_42_ vs. [Cu(HS)(H_2_O)] and [Cu(HS)(H_2_S)]·(H_2_O)_42_ vs. [Cu(HS)(H_2_S)]), simple complexes significantly preferred enrichment-heavy isotopes (Figure 5A). 

For the 13 Cu(II) complex species, at the temperature range of 0~500 °C, the ranges of the Cu isotope fractionation factors for the systems [Cu(NO_3_)_2_]·(H_2_O)_42_ vs. [Cu(NO_3_)_2_], [Cu(NO_3_)(H_2_O)_4_]^+^·(H_2_O)_42_ vs. [Cu(NO_3_)(H_2_O)_4_]^+^, [CuCN]^+^·(H_2_O)_42_ vs. [CuCN]^+^, [Cu(H_2_O)_6_]^2+^·(H_2_O)_42_ vs. [Cu(H_2_O)_6_]^2+^, [CuCl]^+^·(H_2_O)_42_ vs. [CuCl]^+^, [CuCl_2_]·(H_2_O)_42_ vs. [CuCl_2_], [CuCl_3_]^−^·(H_2_O)_42_ vs. [CuCl_3_]^−^, [CuBr_2_]·(H_2_O)_42_ vs. [CuBr_2_], [Cu(OH)_2_]·(H_2_O)_42_ vs. [Cu(OH)_2_], [Cu(COOHCOO)]^+^·(H_2_O)_42_ vs. [Cu(COOHCOO)]^+^, [Cu(CH_3_CH_2_COO)]^+^·(H_2_O)_42_ vs. [Cu(CH_3_CH_2_COO)]^+^, [Cu(HOC_6_H_4_COO)]^+^·(H_2_O)_42_ vs. [Cu(HOC_6_H_4_COO)]^+^, and [Cu(SO_4_)(H_2_O)_3_]·(H_2_O)_42_ vs. [Cu(SO_4_)(H_2_O)_3_] are 0.53~0.06, 0.09~0.02, 0.26~0.04 and 0.49~0.07, 3.92~0.53,0.52~0.06, 0.13~0.02, 0.68~0.10, 1.09~0.23, 3.48~0.47, 3.30~0.45, 2.05~0.27, and 0.52~0.07(‰) (Table 5). That is, when the solvation effect is considered, the Cu isotope fractionation effect of different Cu-bearing species will change obviously. Therefore, the solvation effect is a factor that must be considered in the theoretical calculation of the Cu isotope fractionation effect of the Cu complexes’ solutions. Specifically, for the Cu(II) inorganic ligand complexes, the [Cu(NO_3_)_2_] phase is more heavily isotopically enriched than [Cu(NO_3_)_2_]·(H_2_O)_42_ (Figure 5B). At 100 °C, the Cu isotope fractionation factor between [Cu(NO_3_)_2_]·(H_2_O)_42_ and [Cu(NO_3_)_2_] is −0.28 (‰). However, the [Cu(NO_3_)(H_2_O)_4_]^+^·(H_2_O)_42_ preferentially enriches the heavy Cu isotope more than the [Cu(NO_3_)(H_2_O)_4_]^+^, but there is little difference in the heavy isotope enrichment capacity between these two phases. At 100 °C, the Cu isotope fractionation factor of the system [Cu(NO_3_)(H_2_O)_4_]^+^·(H_2_O)_42_ vs. [Cu(NO_3_)(H_2_O)_4_]^+^ is 0.06 (‰). The Cu isotope fractionation factor of system [CuCN]^+^·(H_2_O)_42_ vs. [CuCN]^+^ is −0.16(‰), that is, [CuCN]^+^·(H_2_O)_42_ is significantly deficient in the heavy Cu isotope compared to [CuCN]^+^. Compared with [Cu(H_2_O)_6_]^2+^, [Cu(H_2_O)_6_]^2+^·(H_2_O)_42_ is a significantly enriched Cu heavy isotope, and the Cu isotope fractionation factor between them is 0.28 (‰) at 100 °C. When the ligands are halogen elements, the solutions tend to be enriched with a heavy Cu isotope compared to simple complexes (Figure 5C). At 100 °C, the Cu isotope fractionation factors of these systems ([CuCl]^+^·(H_2_O)_42_ vs. [CuCl]^+^, [CuCl_2_]·(H_2_O)_42_ vs. [CuCl_2_], [CuCl_3_]^−^·(H_2_O)_42_ vs. [CuCl_3_]^−^, and [CuBr_2_]·(H_2_O)_42_ vs. [CuBr_2_]) are 2.18, 0.27, 0.08, and 0.38 (‰), respectively. Compared with [Cu(OH)_2_], [Cu(OH)_2_]·(H_2_O)_42_ is significantly deficient in heavy isotopes, and the Cu isotope fractionation factor between them is −0.76 (‰) at 100 °C. Cu(SO_4_)(H_2_O)_3_]·(H_2_O)_42_ has a stronger ability to enrich a heavy Cu isotope than [Cu(SO_4_)(H_2_O)_3_], and the Cu isotope fractionation factor between these two species is 0.29 (‰) at 100 °C. For the three organic ligand complexes involved in this study, when the solvation effect is considered, the solutions are significantly enriched with a heavy isotope compared with the simple complexes, and the ability to enrich heavy isotopes is much greater than that of the simple complexes (Figure 5D). Taking the Cu isotope fractionation factors between complex solutions and simple complexes at 100 °C as an example, the Cu isotope fractionation factors of these systems ([Cu(COOHCOO)]^+^·(H_2_O)_42_ vs. [Cu(COOHCOO)]^+^, [Cu(CH_3_CH_2_COO)]^+^·(H_2_O)_42_ vs. [Cu(CH_3_CH_2_COO)]^+^, and [Cu(HOC_6_H_4_COO)]^+^·(H_2_O)_42_ vs. [Cu(HOC_6_H_4_COO)]^+^) are 1.95, 1.84, and 1.12 (‰), respectively (see Table 5). To facilitate other researchers interested in this topic to obtain the Cu isotopic fractionation factors between these complex solutions and simple complexes at any temperature (0–500 °C), the exponential expansions of the Cu isotope fractionation factors of these systems are shown in Table S2 of the Supplementary Materials.

(4) Cu isotope fractionation factors (Cu(I) and Cu(II)) between different Cu-bearing complex solutions

For Cu(I) complex solutions, [CuCl]·(H_2_O)_42_ was selected as the reference material to facilitate the discussion of the magnitude of Cu isotope fractionation factors between different Cu(I)-bearing substances. In the temperature range of 0–500 °C, the study found that the Cu isotope fractionation factors of Cu isotopes of these systems ([CuCl]·(H_2_O)_42_ vs. [CuCl_2_]^−^·(H_2_O)_42_, [CuCl]·(H_2_O)_42_ vs. [CuCl_3_]^2−^·(H_2_O)_42_, [CuCl]·(H_2_O)_42_ vs. [Cu(HS)_2_]^−^·(H_2_O)_42_, [CuCl]·(H_2_O)_42_ vs. [Cu(HS)(H_2_O)]·(H_2_O)_42_ and [CuCl]·(H_2_O)_42_ vs. [Cu(HS)(H_2_S)]·(H_2_O)_42_) vary from 0.11~0.02, 0.52~0.08, −0.63~−0.08, −0.33~−0.04, and −0.32~−0.04 (‰). From these Cu isotope fractionation data, it can be seen that the ^65^Cu enrichment capacity of complex solutions with a Cl^−^ ligand is weaker than that with HS^−^ as ligand. The Cu isotope fractionation factors of systems [CuCl]·(H_2_O)_42_ vs. [CuCl_2_]^−^·(H_2_O)_42_ and [CuCl]·(H_2_O)_42_ vs. [CuCl_3_]^2−^·(H_2_O)_42_ are positive, indicating that [CuCl]·(H_2_O)_42_ is preferentially enriched with a heavy Cu isotope (^65^Cu) (Figure 6A. At 100 °C, the Cu isotope fractionation factors of these two systems are 0.07 and 0.30 (‰), respectively. In other words, [CuCl_3_]^2−^·(H_2_O)_42_ is the system that preferentially enriches a light Cu isotope (^63^Cu). At the same temperature condition, the Cu isotope fractionation factors between [CuCl]·(H_2_O)_42_ and HS^−^-bearing complex solutions ([CuCl]·(H_2_O)_42_ vs. [Cu(HS)_2_]^−^·(H_2_O)_42_, [CuCl]·(H_2_O)_42_ vs. [Cu(HS)(H_2_O)]·(H_2_O)_42_, and [CuCl]·(H_2_O)_42_ vs. [Cu(HS)(H_2_S)]·(H_2_O)_42_) are −0.34, −0.18, and −0.17 (‰), respectively.

For Cu(II) complex solutions, due to the large number of systems involved, to facilitate data analysis and statistics, these systems are divided into three categories according to different ligand types: inorganic ligand complex solutions (NO_3_^−^, CN^−^, H_2_O, OH^−^, and SO_4_^2−^), complex solutions with halogen elements as ligands, and organic ligand complex solutions. For the inorganic ligand complex solutions, [Cu(NO_3_)_2_]·(H_2_O)_42_ was selected as the reference material to discuss the Cu isotope fractionation effect between [Cu(NO_3_)_2_]·(H_2_O)_42_ and other complex solutions. Cu isotope fractionation factors between other phases can also be obtained according to this method. For the sake of article length, I will not list them here. It can be seen from the results that the ability to enrich a heavy isotope for a complex solution with a NO_3_^−^ ligand is stronger than other inorganic ligand complex solutions. At 100 °C, the Cu isotope fractionation factors of these systems ([Cu(NO_3_)_2_]·(H_2_O)_42_ vs. [CuNO_3_(H_2_O)_4_]^+^·(H_2_O)_42_, [Cu(NO_3_)_2_]·(H_2_O)_42_ vs. [CuCN]^+^·(H_2_O)_42_, [Cu(NO_3_)_2_]·(H_2_O)_42_ vs. [Cu(H_2_O)_6_]^2+^·(H_2_O)_42_, [Cu(NO_3_)_2_]·(H_2_O)_42_ vs. [Cu(OH)_2_]·(H_2_O)_42_, and [Cu(NO_3_)_2_]·(H_2_O)_42_ vs. [CuSO_4_(H_2_O)_3_]·(H_2_O)_42_) are 0.12, 0.23, −0.05, 0.03, and 0.04 (‰). These data size relationships indicate that for inorganic ligand complex solutions, [Cu(H_2_O)_6_]^2+^·(H_2_O)_42_, [Cu(OH)_2_]·(H_2_O)_42_, [CuSO_4_(H_2_O)_3_]·(H_2_O)_42_, and [Cu(NO_3_)_2_]·(H_2_O)_42_ have a slightly different ability to enrich heavy isotopes. This phenomenon can be seen more directly in Figure 6B. From Figure 6B, we can see that the maximum variation range of Cu isotope fractionation factors between [Cu(NO_3_)_2_]·(H_2_O)_42_ and the other three substances is only −0.05~0.04(‰). However, the complex solution with a CN^−^ ligand has a special preference for a light Cu isotope (^63^Cu). 

When the ligands of complex solutions are halogen elements, the reference material is [CuCl]^+^·(H_2_O)_42_. According to the study, at 100 °C, the Cu isotope fractionation factors of these isotope exchange pairs ([CuCl]^+^·(H_2_O)_42_ vs. [CuCl_2_]·(H_2_O)_42_, [CuCl]^+^·(H_2_O)_42_ vs. [CuCl_3_]^−^·(H_2_O)_42_, and [CuCl]^+^·(H_2_O)_42_ vs. [CuBr_2_]·(H_2_O)_42_) are 0.34, 0.76, and 0.63 (‰), respectively (Table 6). When the ligand is Cl-, as the number of Cl atoms gradually increases, its ability to enrich heavy isotopes gradually decreases, which can be clearly seen from the variation of the Cu isotope fractionation factors with temperature (Figure 6C). When the complex solutions have the same spatial structure but the ligands are different (such as [CuCl_2_]·(H_2_O)_42_ and [CuBr_2_]·(H_2_O)_42_), the enrichment capacity of light or heavy isotopes is also different. To put it simply, when the ligands are elements of the same main group, with the gradual increase in atomic weight, the enrichment ability of heavy isotopes for the complex solutions with the same coordination structures gradually decreases. This law is the most common consensus of mass-dependent fractionation, and the results of this study also conform to it. 

When ligands are organic ligands, [Cu(COOHCOO)]^+^·(H_2_O)_42_ was chosen as the reference material to discuss the Cu isotope fractionation effect between [Cu(COOHCOO)]^+^·(H_2_O)_42_ and other organic complex solutions. Compared with the Cu isotope fractionation effect between the complex solutions with inorganic ligands, the Cu isotope fractionation effect between the three organic ligand complex solutions involved is significantly smaller. At 100 °C, the Cu isotope fractionation factors of these systems ([Cu(COOHCOO)]^+^·(H_2_O)_42_ vs. [Cu(CH_3_CH_2_COO)]^+^·(H_2_O)_42_ and [Cu(COOHCOO)]^+^·(H_2_O)_42_ vs. [Cu(HOC_6_H_4_COO)]^+^·(H_2_O)_42_) are −0.06 and −0.08 (‰), respectively (Table 6). This also points to the fact that the isotope fractionation between different organic ligand complex solutions is very small (Figure 6D). In order to obtain Cu isotope fractionation factors at different temperatures more conveniently, polynomial expansion coefficients of these Cu isotope fractionation factors between different Cu-bearing organic complex solutions are listed in Table S3 of the Supplementary Materials.

(5) Implication for Cu isotope fractionation in different systems.

At one time, the accepted academic consensus was that the oxidation state of Cu(II) in seawater was important. Non-equilibrium processes, such as biochemical, photochemical, or thermochemical processes occurring in seawater systems, may also promote the formation of Cu(I) [26]. This is also the original starting point for studying the isotope fractionation effect of Cu(I) and Cu(II) complexes. In the natural water body, the existence form and concentration of trace metal elements depend on various competitive processes between the same type of metal ions (such as Cu, Ag, Zn, etc.), and there will be a trade-off between each other. At the same time, the different ligand types will also affect the migration performance of metal elements, such as solubility, mobility, and so on. The complexation and chelation between ligands and Cu(I)/Cu(II) will have an important effect on the existence of metal ions. One of the most intuitive examples is the process of metal ion flow into the ocean with river water, and various physical and chemical processes that occur at the estuary will significantly affect the existence of Cu(II) species [27]. At the estuary, the river water meets the sea water, and there will be obvious estuarine chemical reactions that will form various types of flocculation products. An important factor affecting Cu flocculation is salinity. Hence, various geochemical behaviors of Cu in estuarine play a crucial role in the geochemical cycle of Cu isotopes [28]. The ab initio method also has been used to study the stability of aqueous solutions of various Cu complexes and has made considerable progress in this field [29]. It is important to study the properties of metallic elements in an aqueous solution system to understand their various geochemical behaviors and help to explain various geochemical processes. The behavior of Cu is more complex than other metallic elements [30]. Dissolved Cu^2+^ in soil and natural water bodies do not exist in a single species but in the form of mixed complexes. The transport mode of metal ions in soil and natural water bodies depends on the nature and concentration of chelating agents present in the environment. 

It is well known that humic acid is a widely existing organic matter in natural water and soil. Although the specific molecular structure of humic acid has not been completely determined, it has been found from the existing research results that some functional groups in the structure of humic acid are benzene ring, carboxyl group, hydroxyl group, etc. [31]. In this study, three organic ligands were selected as examples to study the isotope effect of Cu complexes formed by metal–organic ligands. Experimentally, it is common sense that the functional groups that play a decisive role in a substance can be determined by the spectrogram of a substance [32]. The species morphology of Cu(II) in solution/hydrothermal solution systems depends on the concentration of Cl^−^ and the pH of the solution system [33]. The results of this study also support this view. The complex reaction between metal ions and ligands under hydrothermal conditions will form various types of new coordination compounds, which have very important potential applications in material science and earth science [1,34]. In the natural environment, the types of ligands are very diverse. Among them, the two ligands that play an important role in the migration of metal ions in the crust are HS^−^ and Cl^−^. The formation of Cu deposits is also closely related to the existence of various ligands. This is because metal chloride or sulfide (sulfur hydride) complexes play a decisive role in the migration of metal ions. This also reflects laterally that the concentration of complexes in various aqueous solutions not only depends on their chemical stability but also has a direct relationship with the concentration of ligands [35]. Various processes, such as chemical equilibria between minerals and chemical equilibria between aqueous substances, are affected by many factors, such as pH, oxidation states, temperature, and pressure [2]. 

Many geological processes, environmental processes, and life processes can produce impressive Cu isotope fractionation. The Cu isotope fractionation will provide a good tool to study the geochemical cycle of Cu. It is found that the Cu isotope composition will have obvious difference during the formation of soil. The research of the Cu isotope fractionation in soil formed by oxidative weathering found that the variation range of δ^65^Cu in the studied area was 0.57~0.44 and the Cu isotope composition was closely related to the oxygen content [3]. Little et al. found in their research on the isotope fractionation of Cu and Zn in the process of extreme chemical weathering that heavy Cu isotopes (^65^Cu) would be prefertively released during weathering under oxidation conditions and that this phenomenon is independent of the form of Cu [36]. Previous researchers also studied the Cu isotope fractionation in porphyry Cu deposits [37]. It was found that the total Cu isotope variation measured in the samples involved in the study ranged from −16.96‰ to 9.98‰ (expressed in the form of δ^65^Cu). An experimental study on the Cu isotope fractionation between Cu(II) aqueous solution and CuS found that the average value of Δ^65^Cu_(Cu(II)aq-CuS)_ at 20 °C was 3.06 ± 0.14‰ [38]. This study concluded that covellite is a Cu(I)S(I) compound and the REDOX state may be an important controlling factor for abiogenic ^65^Cu/^63^Cu differentiation in low-temperature geological environments [38]. 

The variation of Cu isotopic composition (δ^65^Cu) of the main terrestrial Cu-bearing sulfides is −1‰~1 ‰ and for stream waters is 1.38‰~1.69‰. In general, the Cu-bearing solutions are more enriched with a heavy Cu isotope (^65^Cu) than the Cu-bearing minerals. The Cu isotope fractionation factors between mine wastewater and Cu-bearing minerals (chalcopyrite and enargite) are 1.43 ± 0.14 and 1.60 ± 0.14, respectively [39]. Given various Cu isotope fractionations found in experiments, Liu et al. used first-principle calculations to systematically study the Cu isotope composition of different types of Cu-bearing minerals. The results showed that the Cu isotope compositions (1000lnβ) of different Cu-bearing minerals would be varied from 1‰ to 7‰ [40]. At the same time, it also has been found that the heavy isotope (^65^Cu) in the complex preferentially enriched during the formation of Cu-bearing complexes and the Δ^65^Cu_complex-free_ value varies between +0.14 and +0.84‰ [41]. Cu isotope fractionation not only occurs between various inorganic substances but also between different organic systems. For example, the study of Cu isotope fractionation in plants found that plants preferentially enrich the light Cu isotope (^63^Cu) compared to the soil in which they grow [42]. 

In conclusion, various processes in nature can lead to Cu isotope fractionation. These key parameters of Cu isotope fractionation obtained in this study will provide important theoretical guidance for explaining the mechanism of Cu isotope fractionation from the molecular level. 

## 4. Methods

(1) Theoretical calculation method

The cornerstone works of theoretical computational geochemistry are Urey (1947) and Bigeleisen and Mayer (1947) [6,8]. Their works make it possible to study geochemical parameters (isotope fractionation factors) from the perspective of quantum chemistry. Since then, many related studies have emerged [7,15,16,17,43,44,45,46]. Nowadays, theoretical computational geochemistry has become an effective technique to obtain isotope fractionation factors. For a simple isotope exchange reaction, the equation is as follows:

(3) C63u(I)Cl+C65u(I)(g)⇌C65u(I)Cl+C63u(I)(g)

In Equation (3), Cu(g) represents an ideal gaseous monatom. The formula for calculating the reduced partition function ratio (FRPFR, β factor) is as follows:

(4) RPFR=β=s65s63∏i3n−6ui63ui65exp−ui63/2exp−ui63/21−exp−ui651−exp−ui63

In Equation (4), “s” represents the symmetric number of a molecule or molecular cluster. All the research systems involved in this study only consider the case where one atom is replaced and the symmetric number does not change before and after. The superscripts 63 and 65 represent two different stable isotopes of Cu, namely ^63^Cu and ^65^Cu, respectively. u_i_ is a function of the harmonic frequency and temperature, and its functional relationship with these two variables is as follows:

(5) $$u_{i}=\frac{h\nu_{i}}{k_{b}T}$$

In Equation (5), v_i_ is the calculated harmonic vibration frequencies of the molecule or molecular cluster, and h, K_b_ and T represent Planck constant, Boltzmann constant, and Kelvin temperature, respectively. When obtaining the parameter α, which is the focus of the geochemical field, its theoretical calculation formula is shown as follows (corresponding to the isotope exchange reaction in Equation (3)):

(6) $$\alpha_{Cu(I)Cl-Cu(I)(g)}=\frac{\beta_{Cu(I)Cl}}{\beta_{Cu(I)(g)}}$$

For most element systems, the isotope fractionation factors produced after isotope exchange reactions are usually small. In most cases, 1000lnα is more commonly used to describe the isotope fractionation factors, i.e.,

(7) $$10^{3}\ln\alpha_{Cu(I)Cl-Cu(I)(g)}=10^{3}\ln\beta_{Cu(I)Cl}-10^{3}\ln\beta_{Cu(I)(g)}$$

It can be seen from the above theoretical derivation that the equilibrium stable isotope fractionation factor can be obtained from molecular level as long as the simple harmonic vibration frequencies of molecule/molecular cluster are obtained. According to the theoretical formula of equilibrium isotope fractionation factor, it can be seen that there is a negative correlation between equilibrium stable isotope fractionation factor and temperature, that is, with the increase in temperature, equilibrium stable isotope fractionation factor decreases gradually.

The servers used for all theoretical calculations in this study are Dell PowerEdge T640 which are produced in China. The calculation software used is Gaussian16 (Revision B.01).

(2) computation details

In the treatment of the solvation effect, the method adopted here was “water-droplet” method. Taking Cu(I)Cl aqueous solution as an example, in the calculation, 6 water molecules were taken as a group and added around Cu(I)Cl in turn until Cu(I)Cl·(H_2_O)_42_ was obtained. After that, the structure with 42 water molecules was used as the final solution structure to calculate the β factor. This research method has been proven to be correct and effective by many studies [16,18]. This is because the isotope fractionation effect is a local effect, and the biggest influence on the isotope fractionation effect is the chemical bond (or hydrogen bond) closest to the target atom [47]. 

One of the most representative works on “water-droplet” method is by Liu and Tossell (2005) [45]. In their work, the B isotope effect between B(OH)_3_ and B(OH)_4_^−^ in seawater was studied with a theoretical calculation base of HF/6-31G* level. In addition, they carefully compared the calculation results obtained with different calculation methods (“water droplet” method and self-consistent reaction field (SCRF) methods), and concluded that “water-droplet” method is a more reasonable method to simulate the solvent effect. In this study, Gaussian16 software package was used for theoretical calculation. Becke three-parameter Lee–Yang–Parr (B3LYP) was the theoretical calculation method used for all structural optimizations and simple harmonic vibration frequency calculations [48,49]. The B3LYP method has been widely used to study isotope fractionation factors between different systems [7,13,17,45]. Schauble (2004) used the theoretical methods and theoretical basis set of B3LYP and 6-31G(d) to investigate normal modes for vibration of tetrahedral [CrO_4_]^2−^ [7]. The theoretical basis set of B3LYP/6-311+g(d) in this study was based on the calculation accuracy and calculation cost. In this study, molecular clusters containing 42 water molecules around the solute were used to simulate the Cu-bearing complex aqueous solutions. Therefore, the theoretical calculation was very time-consuming, and the most time-consuming was the process of the geometry optimization. However, once a large enough molecular cluster is optimized, the more accurate isotope fractionation results will be obtained. In this sense, B3LYP/6-311+g(d) is a more compromised basis set. The core reason why this is possible is that the isotope fractionation factor (α) is the ratio of the β values of two different substances. This is also why comparing the β values of different theoretical calculation methods is of no theoretical or practical significance. This point can be confirmed by Equation (6).

## 5. Conclusions

In this study, the equilibrium Cu isotope fractionation effects of different Cu(I)-bearing and Cu(II)-bearing solution/hydrothermal solution systems have been systematically studied using the ab initio calculation method based on first principles. It was found that the Cu isotope enrichment ability of [CuCl2]^−^·(H_2_O)_42_ was significantly different from that of [Cu(HS)_2_]^−^·(H_2_O)_42_. In the temperature range of 0~500 °C, the 1000lnβ of these two species varied from 2.09~0.27 and 2.82~0.37 (‰), respectively. In addition, the type of ligands and the spatial structure of the complexes have significant effects on the equilibrium Cu isotope fractionation factors. The effect of Cu isotope fractionation effects between different Cu-bearing inorganic complexes and between different Cu-bearing organic complexes are quite different. At the same time, the solvation effect is also a key factor that must be considered in the theoretical calculation of the isotope effect between different Cu-bearing solution species. 

The difference in the heavy Cu isotope enrichment capacity between Cu(I) and Cu(II) complex aqueous solutions is very significant. At 150 °C, the variation range of the 100lnβ of Cu (I) and Cu (II) complex aqueous solutions is 0.70~1.21 and 1.75~2.56, respectively, and the difference between the two is relatively large. It also shows that the REDOX process has a significant effect on the fractionation of Cu isotopes, especially on the Cu isotope fractionation in ore-forming fluids. The acquisition of these key equilibrium Cu isotope fractionation parameters will provide necessary theoretical support for a better understanding of the Cu geochemical cycle.

## Data Availability

Data are contained within the article.

## Supplementary Materials

The following supporting information can be downloaded at: https://www.mdpi.com/article/10.3390/molecules29112582/s1, Table S1. The list of energy of optimization results for Cu-bearing complexes with different ligands, where A, B, C, and D represent the results of four parallel theoretical calculations respectively. Table S2. A list of constants of exponential expansions of the Cu isotope fractionation factors between different complex solutions and simple complexes. Table S3. The coefficients of Polynomial expansion of the Cu isotope fractionation factors between different Cu-bearing complex solutions.
