# Supplementary material for: The Theoretical Calculation of the Cu Isotope Fractionation Effect in Solution/Hydrothermal Solution Systems
Source: Molecules. 2024 May 30;29(11):2582. doi: 10.3390/molecules29112582 (PMC11173797; doi:10.3390/molecules29112582)
Supplement: Supplementary file 1 [file molecules-29-02582-s001.zip › molecules-2988883-supplementary.pdf]

Table S1. The list of energy of optimization results for Cu-bearing complexes with different ligands, where A, B, C, and D represent the results of four parallel theoretical calculations respectively.

Energy / Species	E(RB3LYP) (A.U.)								
**Cu(I)**	**$0H_2O$**	**number**	**$6H_2O$**	**$12H_2O$**	**$18H_2O$**	**$24H_2O$**	**$30H_2O$**	**$36H_2O$**	**$42H_2O$**
$[CuCl]^0.nH_2O$	-2100.76	A	-2559.57	-3018.36	-3477.15	-3935.93	-4394.76	-4853.54	-5312.34
		B	-2559.58	-3018.36	-3477.15	-3935.94	-4394.74	-4853.52	-5312.32
		C	-2559.57	-3018.37	-3477.17	-3935.95	-4394.74	-4853.56	-5312.34
		D	-2559.57	-3018.36	-3477.17	-3935.96	-4394.74	-4853.53	-5312.35
$[CuCl_2]^-.nH_2O$	-2561.17	A	-3019.96	-3478.74	-3937.53	-4396.32	-4855.13	-5313.92	-5772.72
		B	-3019.96	-3478.73	-3937.53	-4396.31	-4855.10	-5313.93	-5772.72
		C	-3019.95	-3478.73	-3937.55	-4396.32	-4855.11	-5313.91	-5772.71
		D	-3019.95	-3478.74	-3937.52	-4396.30	-4855.11	-5313.90	-5772.70
$[CuCl_3]^{2-}.nH_2O$	-3021.37	A	-3480.21	-3939.03	-4397.82	-4856.61	-5315.41	-5774.23	-6233.04
		B	-3480.21	-3939.02	-4397.82	-4856.60	-5315.41	-5774.22	-6233.02
		C	-3480.22	-3939.02	-4397.82	-4856.59	-5315.41	-5774.23	-6233.02
		D	-3480.21	-3939.00	-4397.81	-4856.62	-5315.41	-5774.21	-6233.03
$[Cu(HS)_2]^-.nH_2O$	-2438.30	A	-2897.07	-3355.86	-3814.65	-4273.44	-4732.22	-5191.03	-5649.85
		B	-2897.07	-3355.86	-3814.65	-4273.44	-4732.24	-5191.03	-5649.82
		C	-2897.08	-3355.86	-3814.66	-4273.43	-4732.25	-5191.04	-5649.83
		D	-2897.07	-3355.87	-3814.65	-4273.45	-4732.24	-5191.05	-5649.85
$[Cu(HS)(H_2O)].nH_2O$	-2115.82	A	-2574.61	-3033.39	-3492.19	-3950.97	-4409.77	-4868.57	-5327.38
		B	-2574.61	-3033.40	-3492.19	-3950.99	-4409.76	-4868.57	-5327.36
		C	-2574.60	-3033.40	-3492.17	-3950.97	-4409.77	-4868.58	-5327.36
		D	-2574.61	-3033.40	-3492.18	-3950.97	-4409.77	-4868.57	-5327.38
$[Cu(HS)(H_2S)].nH_2O$	-2438.77	A	-2897.56	-3356.34	-3815.11	-4273.91	-4732.72	-5191.52	-5650.31
		B	-2897.56	-3356.36	-3815.14	-4273.92	-4732.72	-5191.53	-5650.34
		C	-2897.55	-3356.31	-3815.14	-4273.94	-4732.74	-5191.53	-5650.32
		D	-2897.58	-3356.35	-3815.13	-4273.95	-4732.73	-5191.52	-5650.32
Cu(II)									
$[Cu(HOC_6H_4COO)]^+.nH_2O$	-2135.45	A	-2594.28	-3053.09	-3511.86	-3970.67	-4429.46	-4888.27	-5347.04
		B	-2594.29	-3053.10	-3511.88	-3970.67	-4429.45	-4888.22	-5347.02
		C	-2594.27	-3053.08	-3511.86	-3970.67	-4429.46	-4888.24	-5347.05
		D	-2594.31	-3053.10	-3511.88	-3970.66	-4429.46	-4888.25	-5347.03
$[Cu(CH_3CH_2COO)]^+.nH_2O$	-1908.07	A	-2366.96	-2825.76	-3284.55	-3743.35	-4202.16	-4660.95	-5119.76
		B	-2366.97	-2825.78	-3284.57	-3743.36	-4202.18	-4660.97	-5119.76
		C	-2366.96	-2825.78	-3284.58	-3743.35	-4202.15	-4660.95	-5119.75
		D	-2366.95	-2825.78	-3284.57	-3743.36	-4202.13	-4660.93	-5119.76
$[Cu(COOHCOO)]^+.nH_2O$	-2018.02	A	-2476.92	-2935.74	-3394.55	-3853.34	-4312.13	-4770.93	-5229.73
		B	-2476.92	-2935.75	-3394.55	-3853.36	-4312.16	-4770.94	-5229.74
		C	-2476.93	-2935.74	-3394.54	-3853.34	-4312.14	-4770.95	-5229.76
		D	-2476.93	-2935.74	-3394.54	-3853.35	-4312.14	-4770.93	-5229.73
$[CuCl]^+.nH_2O$	-2100.39	A	-2559.33	-3018.13	-3476.95	-3935.74	-4394.52	-4853.32	-5312.11
		B	-2559.33	-3018.14	-3476.93	-3935.74	-4394.53	-4853.31	-5312.13
		C	-2559.33	-3018.15	-3476.94	-3935.73	-4394.53	-4853.35	-5312.12
		D	-2559.33	-3018.14	-3476.93	-3935.72	-4394.53	-4853.32	-5312.12
$[CuCl_2].nH_2O$	-2561.02	A	-3019.82	-3478.61	-3937.41	-4396.19	-4855.00	-5313.79	-5772.59
		B	-3019.82	-3478.61	-3937.40	-4396.20	-4854.99	-5313.79	-5772.58
		C	-3019.82	-3478.61	-3937.39	-4396.20	-4854.99	-5313.78	-5772.59
		D	-3019.83	-3478.60	-3937.40	-4396.19	-4854.98	-5313.77	-5772.58
$[CuCl_3]^-.nH_2O$	-3021.41	A	-3480.19	-3938.98	-4397.78	-4856.59	-5315.37	-5774.15	-6232.96
		B	-3480.18	-3938.97	-4397.78	-4856.58	-5315.36	-5774.15	-6232.96
		C	-3480.19	-3938.99	-4397.78	-4856.57	-5315.36	-5774.13	-6232.93
		D	-3480.19	-3938.99	-4397.77	-4856.55	-5315.35	-5774.17	-6232.96
$[Cu(CN)(H_2O)_4]^+.nH_2O$	-2038.97	A	-2497.80	-2956.61	-3415.41	-3874.21	-4333.00	-4791.80	-5250.61
		B	-2497.80	-2956.61	-3415.41	-3874.21	-4332.99	-4791.80	-5250.61
		C	-2497.80	-2956.62	-3415.42	-3874.20	-4333.01	-4791.81	-5250.62
		D	-2497.81	-2956.61	-3415.41	-3874.21	-4333.00	-4791.80	-5250.59
$[Cu(H_2O)_6]^{2+}.nH_2O$	-2098.68	A	-2557.56	-3016.38	-3475.20	-3933.99	-4392.80	-4851.61	-5310.40
		B	-2557.56	-3016.38	-3475.19	-3934.00	-4392.78	-4851.61	-5310.41
		C	-2557.56	-3016.38	-3475.20	-3934.01	-4392.80	-4851.60	-5310.40
		D	-2557.56	-3016.38	-3475.19	-3934.00	-4392.80	-4851.62	-5310.40
$[CuNO_3(H_2O)_4]^+.nH_2O$	-2226.52	A	-2685.34	-3144.15	-3602.95	-4061.75	-4520.55	-4979.34	-5438.14
		B	-2685.35	-3144.15	-3602.94	-4061.74	-4520.54	-4979.32	-5438.14
		C	-2685.35	-3144.15	-3602.95	-4061.75	-4520.54	-4979.33	-5438.13
		D	-2685.35	-3144.13	-3602.94	-4061.73	-4520.51	-4979.32	-5438.12
$[Cu(NO_3)_2].nH_2O$	-2201.31	A	-2660.10	-3118.89	-3577.69	-4036.47	-4495.28	-4954.07	-5412.88
		B	-2660.11	-3118.89	-3577.69	-4036.48	-4495.28	-4954.09	-5412.89
		C	-2660.11	-3118.88	-3577.68	-4036.49	-4495.28	-4954.07	-5412.86
		D	-2660.09	-3118.89	-3577.67	-4036.47	-4495.26	-4954.06	-5412.86
$[Cu(OH)_2].nH_2O$	-1792.20	A	-2251.00	-2709.79	-3168.57	-3627.39	-4086.19	-4544.98	-5003.77

		B	-2251.00	-2709.81	-3168.58	-3627.38	-4086.19	-4544.97	-5003.77
		C	-2251.01	-2709.79	-3168.59	-3627.37	-4086.17	-4545.00	-5003.79
		D	-2250.99	-2709.80	-3168.61	-3627.39	-4086.19	-4544.98	-5003.77
	-6788.88	A	-7247.67	-7706.46	-8165.23	-8624.03	-9082.82	-9541.63	-10000.43
$[CuBr_2].nH_2O$		B	-7247.67	-7706.46	-8165.25	-8624.05	-9082.85	-9541.65	-10000.43
		C	-7247.66	-7706.46	-8165.25	-8624.04	-9082.83	-9541.63	-10000.44
		D	-7247.67	-7706.45	-8165.24	-8624.03	-9082.84	-9541.63	-10000.43
	-2569.04	A	-3027.87	-3486.68	-3945.46	-4404.25	-4863.06	-5321.86	-5780.65
$[CuSO_4(H_2O)_3].nH_2O$		B	-3027.87	-3486.68	-3945.47	-4404.27	-4863.06	-5321.87	-5780.66
		C	-3027.87	-3486.67	-3945.47	-4404.26	-4863.06	-5321.83	-5780.65
		D	-3027.87	-3486.66	-3945.47	-4404.26	-4863.06	-5321.88	-5780.67

Table S2. A list of constants of exponential expansions of the Cu isotope fractionation factors between different complex solutions and simple complexes.

Species \ Coefficient	a	b	c	d
**Cu(I)**				
$[CuCl].(H_2O)_{42}$ vs $[CuCl]$	4.3049×10^{-6}	-1.4342×10^{-4}	2.9193×10^{-2}	5.8662×10^{-4}
$[CuCl_2]^-.(H_2O)_{42}$ vs $[CuCl_2]^-$	-1.1285×10^{-6}	4.1799×10^{-4}	-7.4803×10^{-2}	2.8931×10^{-4}
$[CuCl_3]^{2-}.(H_2O)_{42}$ vs $[CuCl_3]^{2-}$	1.6910×10^{-6}	-2.2440×10^{-4}	5.1957×10^{-2}	2.1353×10^{-4}
$[Cu(HS)_2]^-.(H_2O)_{42}$ vs $[Cu(HS)_2]^-$	6.0422×10^{-7}	1.8859×10^{-5}	-5.1225×10^{-3}	1.3335×10^{-4}
$[Cu(HS)(H_2O)].(H_2O)_{42}$ vs $[Cu(HS)(H_2O)]$	-4.4442×10^{-6}	7.1099×10^{-4}	-6.6132×10^{-2}	3.2122×10^{-4}
$[Cu(HS)(H_2S)].(H_2O)_{42}$ vs $[Cu(HS)(H_2S)]$	-1.2711×10^{-6}	2.7289×10^{-4}	-1.8118×10^{-2}	2.1496×10^{-4}
**Cu(II)**				
$[Cu(NO_3)_2].(H_2O)_{42}$ vs $[Cu(NO_3)_2]$	7.5787×10^{-6}	-1.5719×10^{-4}	-3.8945×10^{-2}	7.7763×10^{-4}
$[Cu(NO_3)(H_2O)_4]^+.(H_2O)_{42}$ vs $[Cu(NO_3)(H_2O)_4]^+$	1.1327×10^{-5}	-4.0478×10^{-4}	1.0113×10^{-2}	1.9331×10^{-3}
$[CuCN]^+.(H_2O)_{42}$ vs $[CuCN]^+$	7.9160×10^{-7}	3.4918×10^{-4}	-2.4328×10^{-2}	1.1451×10^{-3}
$[Cu(H_2O)_6]^{2+}.(H_2O)_{42}$ vs $[Cu(H_2O)_6]^{2+}$	1.9333×10^{-5}	-8.6581×10^{-4}	4.4330×10^{-2}	2.6053×10^{-3}
$[CuCl]^+.(H_2O)_{42}$ vs $[CuCl]^+$	3.2851×10^{-5}	-2.5023×10^{-3}	3.1995×10^{-1}	2.6492×10^{-3}
$[CuCl_2].(H_2O)_{42}$ vs $[CuCl_2]$	5.3809×10^{-6}	2.0509×10^{-4}	3.4941×10^{-2}	1.9346×10^{-3}
$[CuCl_3]^-.(H_2O)_{42}$ vs $[CuCl_3]^-$	1.0059×10^{-5}	-4.2602×10^{-4}	1.3650×10^{-2}	1.4835×10^{-3}
$[CuBr_2].(H_2O)_{42}$ vs $[CuBr_2]$	1.6484×10^{-5}	-7.3738×10^{-4}	5.7243×10^{-2}	1.9979×10^{-3}
$[Cu(OH)_2].(H_2O)_{42}$ vs $[Cu(OH)_2]$	-9.7739×10^{-5}	5.9968×10^{-3}	-1.4397×10^{-1}	-1.8793×10^{-3}
$[Cu(COOHCOOH)]^+.(H_2O)_{42}$ vs $[Cu(COOHCOOH)]^+$	2.9721×10^{-5}	-2.4250×10^{-3}	2.8662×10^{-1}	2.1586×10^{-3}
$[Cu(CH_3CH_2COO)]^+.(H_2O)_{42}$ vs $[Cu(CH_3CH_2COO)]^+$	2.8468×10^{-5}	-2.2656×10^{-3}	2.7118×10^{-1}	2.4653×10^{-3}
$[Cu(HOC_6H_4COO)]^+.(H_2O)_{42}$ vs $[Cu(HOC_6H_4COO)]^+$	2.3739×10^{-6}	-5.3047×10^{-4}	1.5973×10^{-1}	7.8466×10^{-4}
$[Cu(SO_4)(H_2O)_3].(H_2O)_{42}$ vs $[Cu(SO_4)(H_2O)_3]$	1.1970×10^{-5}	-6.2818×10^{-4}	4.4654×10^{-2}	1.2583×10^{-3}

Table S3. The coefficients of Polynomial expansion of the Cu isotope fractionation factors between different Cu-bearing complex solutions.

Species \ Coefficients	a	b	c	d
**Cu(I)**				
$[CuCl].(H_2O)_{42}$ vs $[CuCl_2]^-.(H_2O)_{42}$	3.9882×10^{-6}	-2.7732×10^{-4}	1.0939×10^{-2}	2.8494×10^{-4}
$[CuCl].(H_2O)_{42}$ vs $[CuCl3]^{2-}.(H_2O)_{42}$	7.1219×10^{-6}	-6.1788×10^{-4}	4.5945×10^{-2}	4.2028×10^{-4}
$[CuCl].(H_2O)_{42}$ vs $[Cu(HS)_2]^-.(H_2O)_{42}$	1.9301×10^{-6}	9.1882×10^{-5}	-4.8272×10^{-2}	3.9616×10^{-4}
$[CuCl].(H_2O)_{42}$ vs $[Cu(HS)(H_2O)].(H_2O)_{42}$	1.0013×10^{-6}	-2.5369×10^{-6}	-2.4743×10^{-2}	9.4938×10^{-5}
$[CuCl].(H_2O)_{42}$ vs $[Cu(HS)(H_2S)].(H_2O)_{42}$	3.3789×10^{-6}	-1.4010×10^{-4}	-2.2503×10^{-2}	3.1016×10^{-4}
Cu(II)				
$[Cu(NO_3)_2].(H_2O)_{42}$ vs $[Cu(NO_3)(H_2O)_4]^+.(H_2O)_{42}$	1.0773×10^{-6}	-1.9450×10^{-4}	1.8620×10^{-2}	-1.5864×10^{-4}
$[Cu(NO_3)_2].(H_2O)_{42}$ vs $[CuCN]^+.(H_2O)_{42}$	7.3303×10^{-6}	-5.8023×10^{-4}	3.5222×10^{-2}	-1.6850×10^{-4}
$[Cu(NO_3)_2].(H_2O)_{42}$ vs $[Cu(H_2O)_6]^{2+}.(H_2O)_{42}$	-2.5870×10^{-6}	1.3257×10^{-4}	-7.4135×10^{-3}	-3.4166×10^{-4}
$[Cu(NO_3)_2].(H_2O)_{42}$ vs $[Cu(OH)_2].(H_2O)_{42}$	-3.9256×10^{-6}	2.4103×10^{-4}	3.0166×10^{-3}	-3.1914×10^{-4}
$[Cu(NO_3)_2].(H_2O)_{42}$ vs $[CuSO_4(H_2O)_3].(H_2O)_{42}$	-3.4415×10^{-6}	1.5330×10^{-4}	4.8729×10^{-3}	-2.5302×10^{-4}
$[CuCl]^+.(H_2O)_{42}$ vs $[CuCl_2].(H_2O)_{42}$	8.6780×10^{-6}	-6.7118×10^{-4}	5.1853×10^{-2}	4.7030×10^{-4}
$[CuCl]^+.(H_2O)_{42}$ vs $[CuCl_3]^-.(H_2O)_{42}$	1.9252×10^{-5}	-1.4222×10^{-3}	1.1427×10^{-1}	1.2759×10^{-3}
$[CuCl]^+.(H_2O)_{42}$ vs $[CuBr_2].(H_2O)_{42}$	1.1806×10^{-5}	-9.8766×10^{-4}	9.3440×10^{-2}	6.9064×10^{-4}
$[Cu(COOHCOO)]^+.(H_2O)_{42}$ vs $[Cu(CH_3CH_2COO)]^+.(H_2O)_{42}$	-1.0811×10^{-6}	8.4619×10^{-5}	-9.3475×10^{-3}	-1.8322×10^{-4}
$[Cu(COOHCOO)]^+.(H_2O)_{42}$ vs $[Cu(HOC_6H_4COO)]^+.(H_2O)_{42}$	3.2759×10^{-6}	-1.9185×10^{-4}	-1.0354×10^{-2}	4.2025×10^{-4}
